# Design, Synthesis and Anti-Proliferative Activities of 2,6-Substituted Thieno[3,2-*d*]pyrimidine Derivatives Containing Electrophilic Warheads

**DOI:** 10.3390/molecules22050788

**Published:** 2017-05-12

**Authors:** Qiumeng Zhang, Zonglong Hu, Qianqian Shen, Yi Chen, Wei Lu

**Affiliations:** 1 School of Chemistry and Molecular Engineering, East China Normal University, 3663 North Zhongshan Road, Shanghai 200062, China; qiumeng-zhang@foxmail.com; 2 Division of Anti-Tumor Pharmacology, State Key Laboratory of Drug Research, Shanghai Institute of Materia Medica, Chinese Academy of Sciences, Shanghai 201203, China; zlhu@jding.dhs.org (Z.H.); shenqianqian@simm.ac.cn (Q.S.)

**Keywords:** thieno[3,2-*d*]pyrimidines, antitumor activities, acrylamide warheads

## Abstract

Thieno[3,2-*d*]pyrimidine as an effective pharmacophore has been extensively studied. However, its 2,6-substituted derivatives are rarely reported. In the present study, eighteen 2,6-substituted thieno[3,2-*d*]pyrimidine derivatives containing electrophilic warheads were designed based on the first known Fibroblast growth factor receptor-4 (FGFR4) inhibitor Blu9931. Unexpectedly, all of the derivatives exhibited negligible activity against FGFR4. However, most of the target compounds exhibited antiproliferative activities against four human cancer cell lines, including A431, NCI-H1975, Ramos and SNU-16. Compound **12** showed the most potent antiproliferative activities on the above four cell lines with IC_50_ values of 1.4 μM, 1.2 μM, 0.6 μM, and 2.6 μM, respectively. Additionally, the antiproliferative activity of **12** against MDA-MB-221 proved that **12** had the selectivity towards certain tumor cell lines. Furthermore, preliminary structure-activity relationship analysis was discussed based on the experimental data.

## 1. Introduction

**Figure 1:**
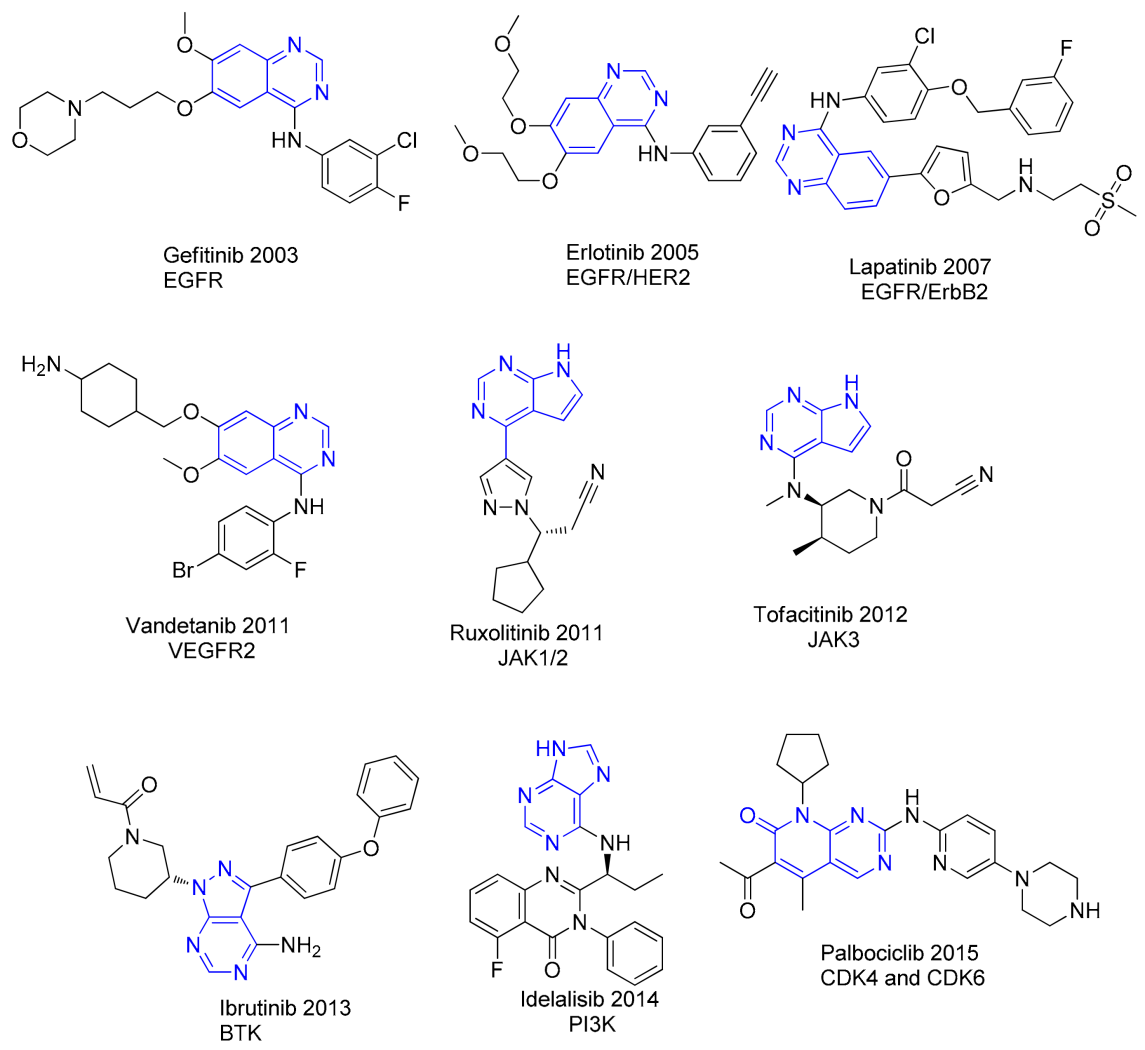
Structures of some Food and Drug Administration (FDA) approved drugs with bicyclic pyrimidine scaffold.

**Figure 2:**
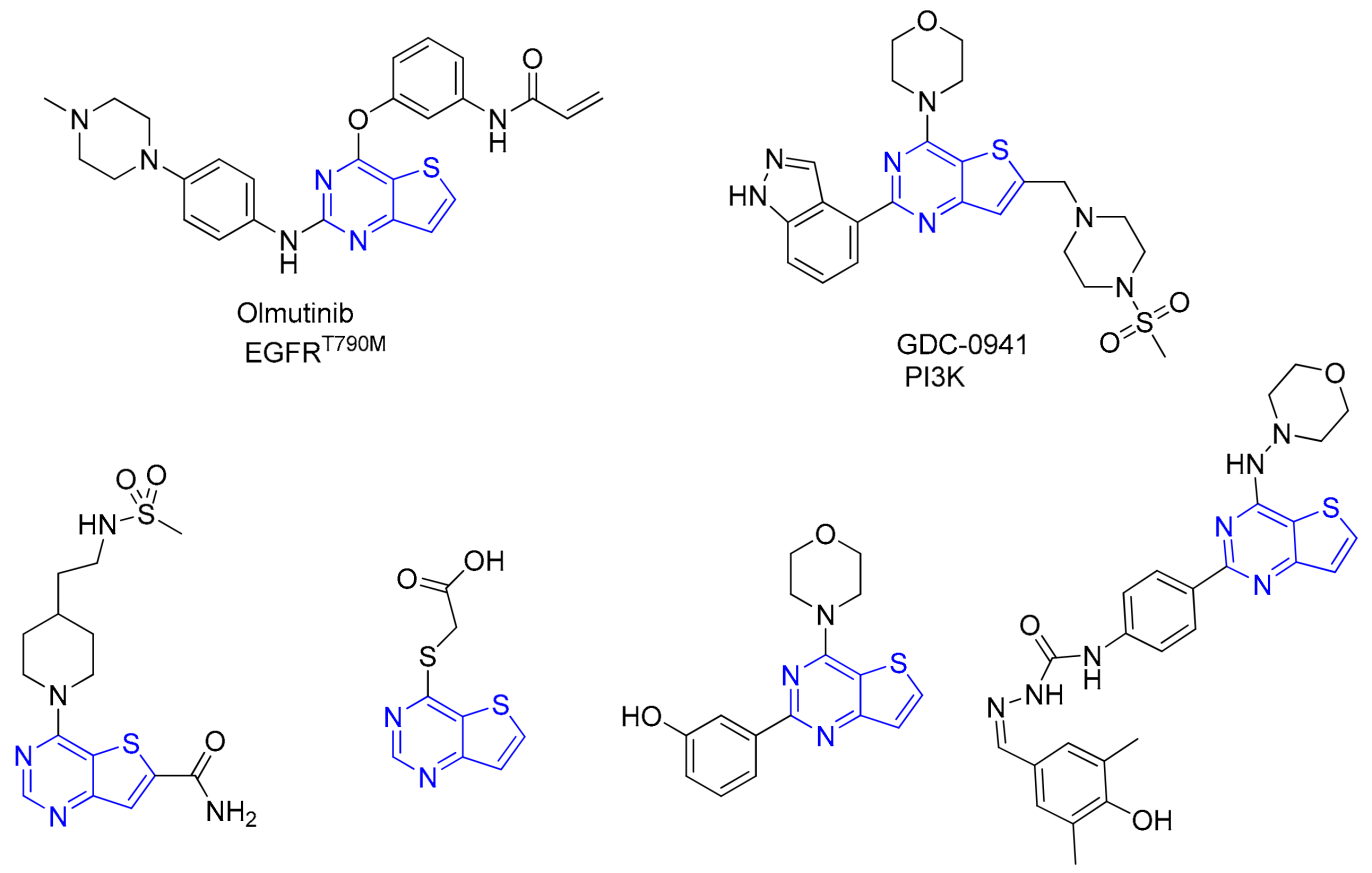
A part of reported thieno[3,2-*d*]pyrimidine structures.

Cancer is one of the most formidable enemies to the public health worldwide [1]. Despite the numerous achievements in the field of cancer pharmacotherapy, there is a continuous demand for novel agents with improved therapeutic efficacy, selectivity, and safety. Over the past few decades, more and more drugs with fused bicyclic pyrimidine scaffolds have been approved by the Food and Drug Administration (FDA) with significant biological activities, especially antitumor activities. These fused bicyclic pyrimidines exhibit anticancer function by targeting different kinases, such as Epidermal growth factor receptor (EGFR), Brutons tyrosine kinase (BTK), Janus Kinase (JAK), and Phosphatidylinositol 3 kinase (PI_3_K) (Figure 1) [2,3,4,5,6]. Considering broader applications of fused bicyclic pyrimidines in anticancer drugs, chemical modifications of the bicyclic pyrimidine scaffolds have been extensively studied. Previously, various thieno[3,2-*d*]pyrimidine derivatives have attracted attention due to their broad spectrum activities, such as GDC-0941 and Olmutinib (Figure 2) [7,8,9,10,11,12,13,14,15,16,17,18]. Although a large number of studies on derivatives of thieno[3,2-*d*]pyrimidine have been documented, the 2,6-substituted analogs are seldom reported.

**Figure 3:**
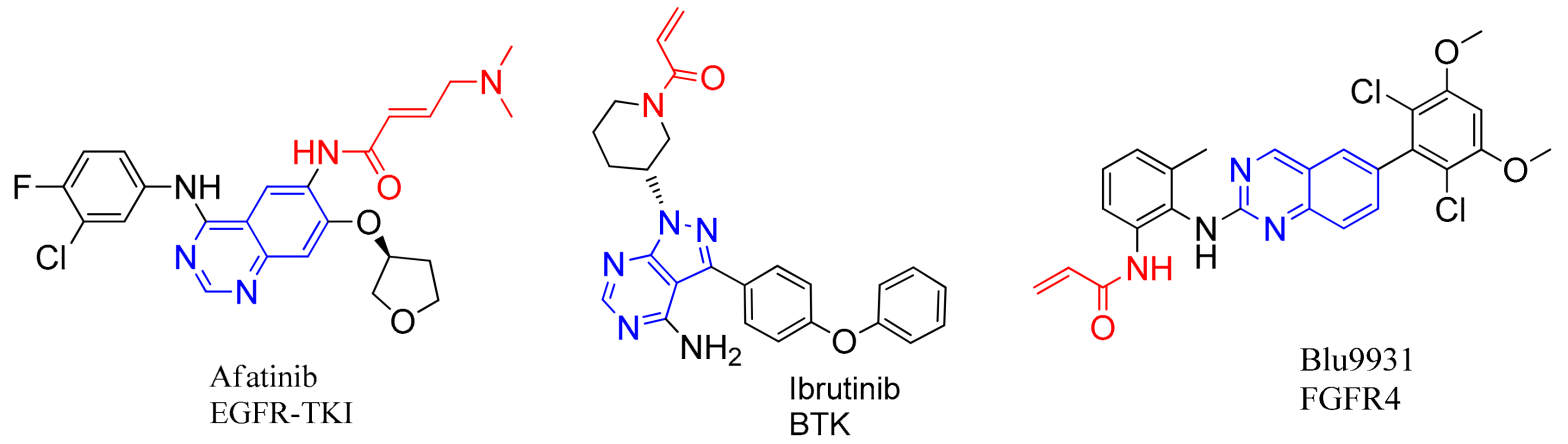
Structures of some covalent inhibitors.

Recently, therapies using covalent drugs have been proved to be successful for various indications. Although there have been concerns about the toxicity of covalent drugs, the high potencies and prolonged effects might result in less frequent drug dosing and wide therapeutic margins for patients [19,20,21,22]. Many covalent drugs have been reported for the treatment of cancer by inhibiting different targets. Among these derivatives, Afatinib, an irreversible EGFR tyrosine kinase inhibitor (TKI) [23], was approved by the FDA for the treatment of metastatic non-small cell lung cancer in 2013 [24]. Ibrutinib, a selective irreversible BTK inhibitor, has been approved by FDA for treatment of patients with chronic lymphocytic leukemia, mantle cell lymphoma and Waldenstrom’s macroglobulinemia [25,26]. Blu9931, which contains a quinazoline core and an acrylamide electrophilic warhead, is the first selective small molecule inhibitor of FGFR4 (Figure 3) [27]. Evidence suggests that covalent drugs are a feasible antitumor strategy. It is worth mentioning that bicyclic pyrimidine scaffolds are widely used in covalent drug design.

**Figure 4:**
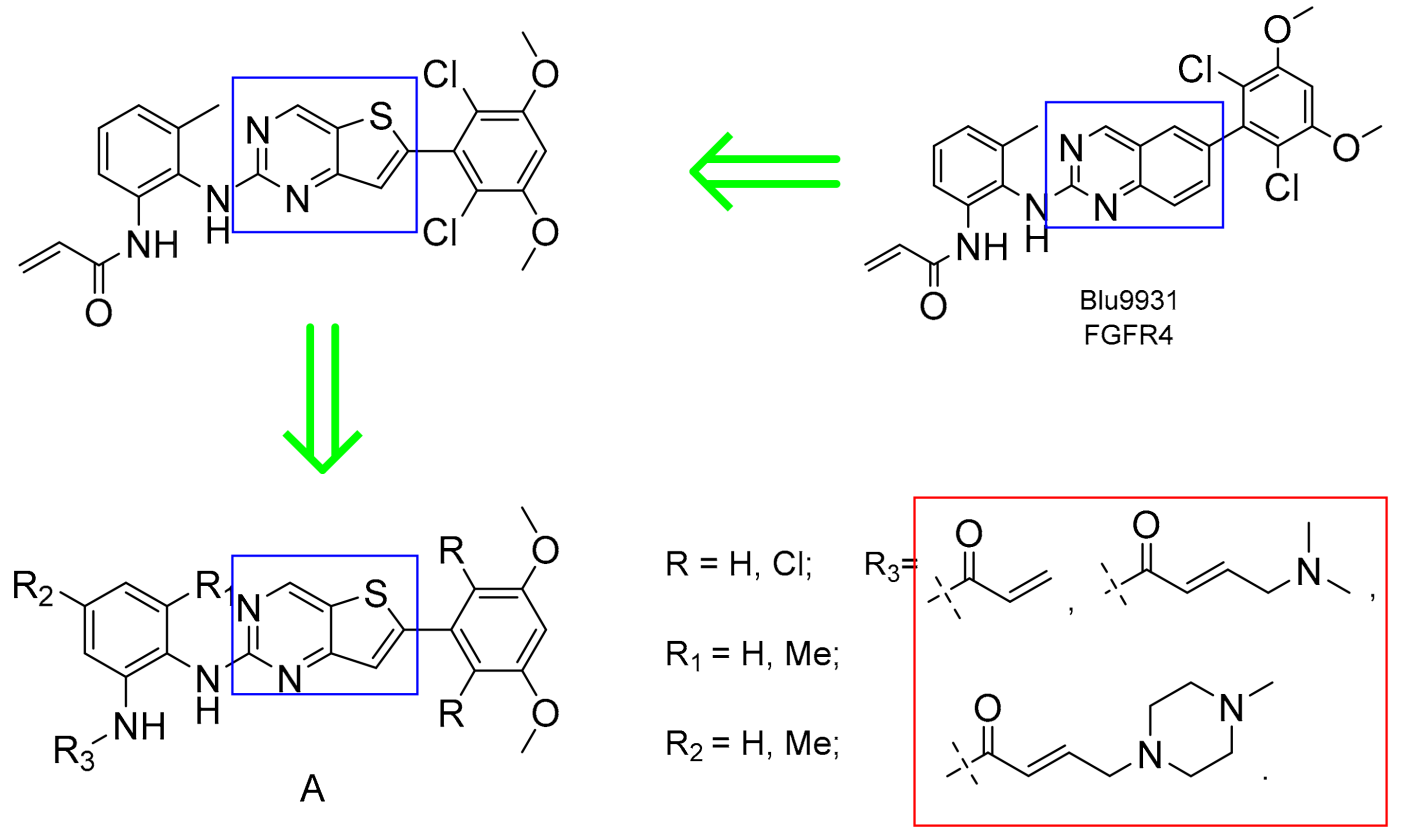
Structures and design strategy for target compounds.

Based on previously published results and our continued interest in studying thieno[3,2-*d*]pyrimidine derivatives, we decided to design a series of 2,6-substituted thieno[3,2-*d*]pyrimidine compounds containing electrophilic warheads based on the first known FGFR4 inhibitor Blu9931. FGFR4 is a tyrosine kinase that regulates a wide range of important biological functions. The fibroblast growth factor 19 (FGF19) is the first FGF member exhibiting selective binding to its receptor FGFR4 [28]. Aberrant signaling through the FGF19/FGFR4 signaling complex has been shown to cause hepatocellsular carcinoma in mice and has indicated a similar role in humans [27]. Therefore, FGFR4 is a potential therapeutic target for anticancer drugs. In addition, FGFR1 is highly homologous to FGFR4. Herein, we presented the design, synthesis and antitumor activities of the compounds related to the general structure A (Figure 4). The preliminary structure-activity relationship was also discussed.

## 2. Results and Discussion

### 2.1. Chemistry

**Scheme 1:**
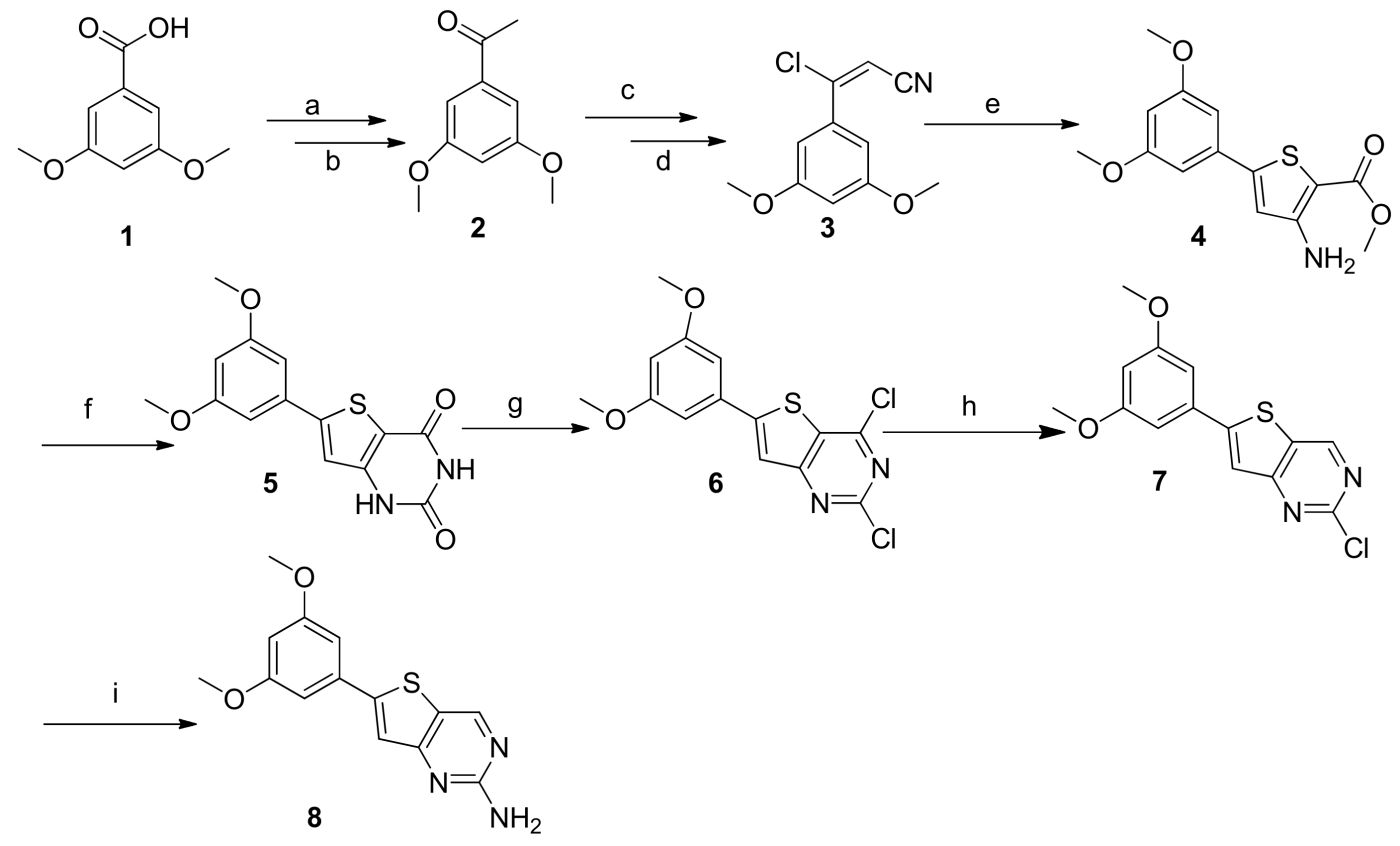
Synthesis of key intermediate **8**. *Reagents and conditions*: (a) i: thionyl chloride, reflux; ii: ethyl potassium malonate, Et_3_N, MgCl_2_, anhydrous MeCN, r.t., overnight; (b) 3 M HCl, EtOH, reflux, 3 h, 79%; (c) POCl_3_, anhydrous *N*,*N*-Dimethylformamide (DMF); (d) hydroxylamine hydrochloride, anhydrous MeCN, r.t., overnight , 40% over two steps; (e) methyl 2-mercaptoacetate, MeONa, anhydrous MeOH, 0–80 °C, 2 h, 96%; (f) urea, neat, 170 °C, 4 h, 89%; (g) POCl_3_, reflux, overnight, 65%; (h) Pd/C, NaHCO_3_, Ethyl acetate (EA), EtOH, r.t., overnight, 79%; (i) 4 M NH_3_/EtOH, 150 °C; 48 h, 67%.

Detailed synthetic strategy to key intermediate **8** was illustrated in Scheme 1. Compound **4** was synthesized according to published procedures [29] and then treated with urea at 170 °C to produce compound **5**. Subsequently, compound **5** was treated with POCl_3_ followed by the treatment with Pd/C/NaHCO_3_ to produce **7**. The newly synthesized **7** was treated with NH_3_/EtOH (4 M) at 150 °C in a steel tube to afford **8**.

**Scheme 2:**
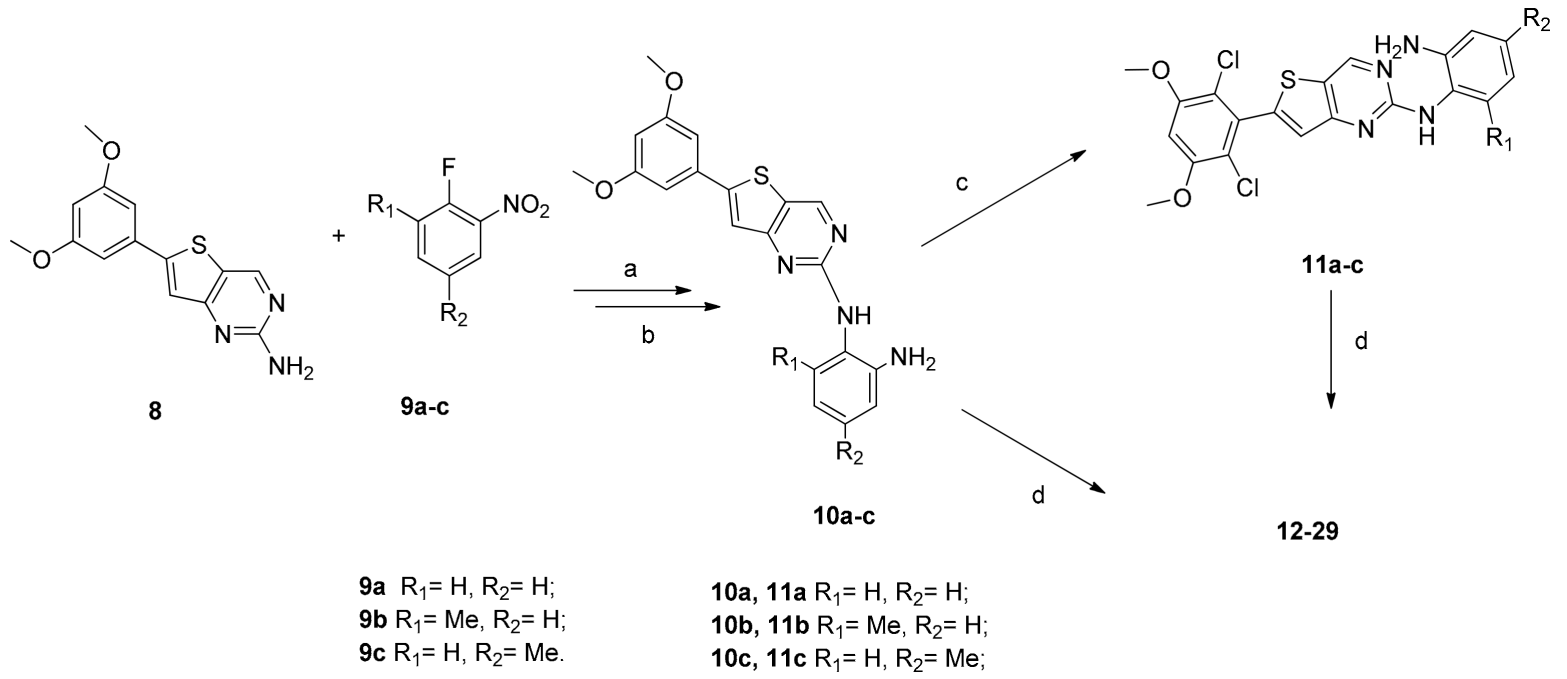
Synthesis of target compounds **12**–**29**. *Reagents and conditions*: (a) NaH, anhydrous *N*,*N*-Dimethylformamide (DMF), r.t.; (b) Fe(OH)_3_, 80% hydrazine hydrate, EtOH, reflux; (**c**) Sulfonyl chloride, Dichloromethane (DCM) or Tetrahydrofuran (THF), −10 °C, 20 min; (d) i: Acryloyl chloride or (*E*)-4-bromobut-2-enoyl chloride, *N*,*N*-Diisopropylethylamine (DIPEA), anhydrous DCM, −10 °C; ii: DMF, NaI, 1-Methylpiperazine or 30% dimethylamine solution in water, r.t.

The synthetic route for the synthesis of derivatives **12**–**29** was depicted in Scheme 2 and the chemical structures of **12**–**29** were shown in Table 1. The derivative **8** was involved in a nucleophilic aromatic substitution reaction in the presence of **9a**–**c** to obtain crude products. Furthermore, reduction of the nitro group was carried out under basic conditions to give the compound **10a**–**c**, which was then treated with sulfonyl chloride to give **11a**–**c**. In addition, **10a**–**c** and **11a**–**c** were mixed with acryloyl chloride to afford the targets **12**–**17**. On the other hand, **10a**–**c** and **11a**–**c** were treated with (*E*)-4-bromobut-2-enoyl chloride before reacting with 1-methylpiperazine or 30% dimethylamine solution in water to afford targets **18**–**29**.

### 2.2. Biological Evaluation

#### 2.2.1. In Vitro Enzymatic Assays–FGFR1 and FGFR4

Firstly, the FGFR1 and FGFR4 enzymatic assays of compounds **12**–**29** were performed, since their structures are similar to the first small molecular FGFR4 inhibitor Blu9931. Unfortunately, all of the compounds lost the enzyme activities against FGFR1 and FGFR4 with less than 50% inhibition at 1 μM. The results summarized in Figures S1 and S2 (supplied in supplementary data) were expressed as inhibition rates, which were derived from double independent determinations (the information of positive controls can be found in supplementary data), suggesting that the interaction between Blu9931 and FGFR4 might be sensitive to the specific angle of two benzene rings.

#### 2.2.2. In Vitro Cytotoxic Activities and SAR Analysis

The antiproliferative activity of compounds **12**–**29** against A431 (human skin squamous cell carcinoma), NCI-H1975 (human lung adenocarcinoma cell), Ramos (human B lymphoma cell) and SNU-16 (human gastric cancer cell) cell lines were tested. Using the approved drug Doxorubicin (ADR) as positive controls, the results of the mean values of experiments from three independent determinations, expressed as half-maximal inhibitory concentration (IC_50_) values, were summarized in Table 2.

As presented in Table 2, most of the target compounds exhibited moderate antiproliferative activities against Ramos cells. The different substituents of hydrogen or methyl in benzene ring were first investigated. Compound **12** exhibited moderate activity (IC_50_ = 0.6 μM), while the replacement of hydrogen with *o*-methyl group (compound **13**) or *p*-methyl group (compound **14**) resulted in a slight loss of activity (IC_50_ = 3.9 μM and 2.7 μM, respectively), suggesting that the antiproliferative activity is sensitive to small changes on the benzene ring adjacent to the pyrimidine side. To further identify the effects of the benzene ring next to the thiophene side on the biological profiles, compounds **15**–**17** were synthesized and tested. Dramatically reduced antitumor activities were observed when **12**–**14** were transformed into their respective dichloride substituted derivatives **15**–**17** (IC_50_ = 8.0 μM, 14.1 μM and 6.4 μM, respectively). The result suggests that the benzene ring next to the thiophene side plays an important role in antitumor activity. In addition, analogues **18**–**29** with different Michael acceptors displayed significantly reduced activity against Ramos cells compared with the corresponding compound with acryloyl group. Most of the compounds **12**–**29** also exhibited moderate to great antiproliferative activity against the other three cell lines. It is interesting to note that the structure-activity relationship analyses of **12**–**29** against the four tested cell lines were highly consistent.

Furthermore, the antiproliferative activity of the most potent compound **12** against NCI-H1581 (Human non-small cell lung cancer cell line), MDA-MB-231 (Human breast cancer cell line) was evaluated. The derivative **12** showed loss of antiproliferative activity against MDA-MB-221 (IC_50_ > 20 μM) indicating that **12** exhibits selectivity towards certain tumor cell lines (Table 3).

#### 2.2.3. In Vitro Enzymatic Assay—BTK

BTK plays a crucial role in B cell lines growth and reproduction [30]. It has been well documented that most of the BTK inhibitors formed a covalent bond between its electrophilic warhead and a nucleophilic center in the BTK protein. As shown in Table 2, most of the target compounds displayed potent anti-proliferative activity on Ramos cells (B cell lines). Therefore, the inhibition of **12**–**29** against BTK at 10 nM, 100 nM and 500 nM were tested. Using Ibrutinib as the positive control, the results of the mean values of experiments from double independent determinations, expressed as inhibition rates, were exhibited in Figure S3 (supplied in Supplementary Data). Compounds **12**–**29** demonstrated less than 40% inhibition against BTK at 500 nM, and their inhibitory activities are much inferior to Ibrutinib. These results indicated that BTK is not the biological target for these compounds.

#### 2.2.4. In Vitro Enzymatic Assays

To investigate the molecular mechanisms, we screened compound **12** against twenty selected tyrosine kinases. The results summarized in Table S3 (supplied in Supplementary Data) were expressed as inhibition rates, derived from three independent determinations (details related to positive controls can be found in Supplementary Data). Unfortunately, compound **12** was judged to have a negligible impact on the tested kinases.

## 3. Materials and Methods

### 3.1. Materials and Methods

All reagents and solvents were purchased from commercial suppliers and were further purified or dried if necessary. The ^1^H-NMR spectra and ^13^C-NMR spectra were recorded for CDCl_3_, MeOD, or Dimethyl sulfoxide-*d*_6_ (DMSO-*d*_6_) by a Bruker DRX-400 (400 MHz) (Bruker Biospin AG, Fällanden, Switzerland) with Tetramethylsilane (TMS) as an internal standard. Chemical shifts were reported as δ (ppm) and spin–spin coupling constants as *J* (Hz) values. The mass spectra were obtained on a Waters -SDQ mass spectrometer (Manchester, UK) or Waters SYNAPT G2 ESI-TOF-MS analyzer. Melting points were determined on an SGW X-4 melting point detector (Shanghai, China), uncorrected and reported in degrees Centigrade. The derivatives synthesized were purified by column chromatography using silica gel (200–300 mesh). The purity of all tested derivatives was established by High Performance Liquid Chromatography (HPLC) to be >95%.

### 3.2. General Synthesis

#### 3.2.1. 1-(3,5-Dimethoxyphenyl)ethanone (**2**)

Step 1. 3,5-dimethoxybenzoyl chloride

3,5-dimethoxybenzoic acid (27.3 g, 150 mmol, compound **1**) was suspended in 120 mL thionyl chloride, and the mixture was heated and held at reflux until **1** dissolved. Then, thionyl chloride was evaporated under reduced pressure conditions and the residue was diluted with 150 mL anhydrous acetonitrile.

Step 2.  Ethyl 3,5-dimethoxybenzoate

Ethyl potassium malonate (51 g, 300 mmol) and magnesium chloride (36 g, 375 mmol) were suspended in 600 mL anhydrous acetonitrile, and 63 mL Et_3_N was added slowly. The mixture stirred at room temperature for 2 h. Then 3,5-dimethoxybenzoyl chloride in 50 mL anhydrous acetonitrile was added dropwise. The reaction mixture stirred at room temperature overnight. Dilute hydrochloric acid added to the mixture until pH < 5. The aqueous layer was extracted three times with ethyl acetate (EA) and the combined organic layers were washed with brine and then dried over anhydrous sodium sulfate, filtered and the solution was concentrated under reduced pressure to give the corresponding crude product.

Step 3.  1-(3,5-dimethoxyphenyl)ethanone (**2**)

The crude product from step 2 dissolved in 150 mL 3 M HCl and 50 mL ethanol. The reaction mixture was heated at reflux for 2 h. After cooling, the solvent was reduced under reduced pressure. The residue was separated on a silica gel column, eluting with a petroleum ether:ethyl acetate (PE/EA) (6:1 *v*/*v*) solvent system to give 2 (21.3 g, 79% over three steps); colorless oil; ^1^H-NMR (400 MHz, CDCl_3_) δ 7.08 (s, 2H), 6.64 (s, 1H), 3.83 (s, 6H), and 2.56 (s, 3H).

#### 3.2.2. (*E*)-3-Chloro-3-(3,5-dimethoxyphenyl)acrylonitrile (**3**)

In addition, 15 mL POCl_3_ was added dropwise into 25 mL anhydrous DMF, the mixture stirred at room temperature for 15 min. Compound **2** (13.8 g, 78 mmol) in 30 mL anhydrous DMF was added to the mixture carefully and stirred for 20 min, and then the reaction was heated to 40 °C for 1 h (monitored by Thin Layer Chromatography, TLC). Then, the mixture was allowed to cool down to 25 °C, and 21.3 g hydroxylamine hydrochloride was added in batches slowly because of the intense heat released. Another 150 mL of anhydrous acetonitrile was added and the mixture stirred at room temperature for overnight. Then, the mixture poured into ice water, stirred for 5 h, filtered, and then the solid was washed by water and ether. The crude product was purified by eluting through a silica gel column with a 1:3 CH_2_Cl_2_/PE solvent system to give **3** (6.96 g, 40% over two steps); colorless oil; ^1^H-NMR (400 MHz, CDCl_3_) δ 6.76 (s, 2H), 6.58 (s, 1H), 6.00 (s, 1H), and 3.83 (s, 6H).

#### 3.2.3. Methyl 3-Amino-5-(3,5-dimethoxyphenyl)thiophene-2-carboxylate (**4**)

Methyl 2-mercaptoacetate (3 mL, 33 mmol) was dissolved in 45 mL MeOH, MeONa (2.4 g, 45 mmol) was added slowly, and the mixture was stirred at 25 °C for half an hour. Compound **3** (6.72 g, 10 mmol) was added and then heated to reflux for 1 h (monitored by TLC), and then the mixture was allowed to cool down to 25 °C and poured into ice water, filtered, and then the filtrate was extracted with EA twice, and the combined organic layers were washed with brine and then dried over anhydrous sodium sulfate, filtered and concentrated. The crude product was purified by eluting through a silica gel column with a 1:8 EA/PE solvent system to give **4** (8.4 g, 96%); yellow oil; ^1^H-NMR (400 MHz, CDCl_3_) δ 6.74 (s, 1H), 6.72 (s, 2H), 6.46 (s, 1H), 5.46 (s, 2H), 3.84 (s, 3H), 3.82 (s, 6H).

#### 3.2.4. 6-(3,5-Dimethoxyphenyl)thieno[3,2-*d*]pyrimidine-2,4(1*H*,3*H*)-dione (**5**)

Compound **4** (8.2 g, 28 mmol) was reacted with 40 g urea at 180 °C for 8 h (monitored by TLC). The mixture was cooled to 120 °C, then added into 500 mL NaOH (1 M) solution, and stirred for 1 h. After filtration, the solid was washed with water and dried by vacuum to obtain **5** (7.6 g, 89%); yellow solid; M.p.: >250 °C; ^1^H-NMR (400 MHz, DMSO-*d*_6_) δ 11.65 (s, 1H), 11.26 (s, 1H), 7.24 (s, 1H), 6.84 (s, 2H), 6.61 (s, 1H), 3.82 (s, 6H); ^13^C-NMR (101 MHz, DMSO-*d*_6_) δ 161.0, 158.8, 157.7, 151.5, 146.6, 134.0, 113.5, 110.0, 104.2, 101.5, 55.5; ESI-MS: *m*/*z* 305.1 [M + H]^+^.

#### 3.2.5. 2,4-Dichloro-6-(3,5-dimethoxyphenyl)thieno[3,2-*d*]pyrimidine (**6**)

Compound **5** (7.5 g, 22 mmol) was dissolved in 30 mL POCl_3_. The solution was heated to reflux for overnight (monitored by TLC). The mixture was allowed to cool down to 40 °C, and then added to 300 mL ice water slowly, white solid precipitated slowly. The precipitate was collected, washed with water and dried to provide the desired product **6** (4.9 g, 65%); white solid; M.p.: >250 °C; ^1^H-NMR (400 MHz, CDCl_3_) δ 7.62 (s, 1H), 6.84 (d, 2H), 6.58 (s, 1H), 3.87 (s, 6H); ^13^C-NMR (101 MHz, CDCl_3_) δ 163.9, 161.5, 157.5, 156.4, 154.7, 133.7, 128.8, 119.4, 105.3, 102.5, 55.6; ESI-MS: *m*/*z* 341.0 [M + H]^+^.

#### 3.2.6. 2-Chloro-6-(3,5-dimethoxyphenyl)thieno[3,2-*d*]pyrimidine (**7**)

To a solution of **6** (4.8 g, 14 mmol) and NaHCO_3_ (2.35 g, 28 mmol) in 20 mL EtOH and 20 mL EA, 10% Pd/C was added (960 mg, 20% by wt). The suspension was stirred at room temperature under an atmosphere of H_2_ for 23 h. A second portion of 10% Pd/C (980 mg, 20% by wt) was added after 12 h. The reaction mixture was filtered through Celite^®^ with EtOAc washings. The filtrate was washed with H_2_O/brine (4:1), dried by MgSO_4_, filtered and concentrated under reduced pressure to provide **7** (3.4 g, 79%); light yellow solid; M.p.: >250 °C; ^1^H-NMR (400 MHz, CDCl_3_) δ 9.04 (s, 1H), 7.62 (s, 1H), 6.87 (s, 2H), 6.58 (s, 1H), 3.88 (s, 6H); ^13^C-NMR (101 MHz, CDCl_3_) δ 163.6, 161.4, 157.7, 157.2, 152.7, 134.1, 129.7, 119.0, 105.4, 102.2, 77.3, 77.0, 76.7, 55.6; ESI-MS: *m*/*z* 307.0 [M + H]^+^.

#### 3.2.7. 6-(3,5-Dimethoxyphenyl)thieno[3,2-*d*]pyrimidin-2-amine (**8**)

Compound **7** (3.1 g, 10 mmol) was dissolved in 80 mL 4 M NH_3_/EtOH. The solution was heated to 150 °C for 48 h in sealed tube. The reaction mixture was cooled to room temperature and then poured into ice water, filtered through Celite^®^ with EtOAc washings. The solid dried by vacuum to obtain compound **8** (1.9 g, 67%); Off-white solid; M.p.: 249.4–250.2 °C; ^1^H-NMR (400 MHz, DMSO-*d*_6_) δ 8.89 (s, 1H), 7.65 (s, 1H), 6.96 (s, 2H), 6.62 (s, 1H), 6.57 (s, 2H), 3.83 (s, 6H); ^13^C-NMR (101 MHz, DMSO-*d*_6_) δ 162.7, 161.8, 160.9 152.8, 152.3, 134.7, 119.5, 119.2, 104.5, 101.5, 55.5; ESI-MS: *m*/*z* 288.1 [M + H]^+^.

#### 3.2.8. General Procedure 1 of the Synthesis of Compounds **10a**–**c**

The solution of compound **8** (287 mg, 1 mmol) and NaH (60%, 80 mg, 2 mmol) in 10 mL anhydrous DMF was stirred for 30 min, corresponding *o*-fluoronitrobenzene (**9a**–**c**, 1.05 mmol) in 5 mL anhydrous DMF was added to the mixture dropwise. The reaction mixture was stirred for overnight and then poured into 100 mL water, filtered through Celite with water washings. The solid dried by vacuum to give corresponding crude products. To a solution of corresponding crude products (0.8 mmol) in 15 mL EtOH, Fe(OH)_3_ (43mg, 0.4 mmol) and Hydrazine hydrate (80%, 0.5 mL, 8 mmol) were added. The mixture was heated to 70 °C for about 4 h (monitored by TLC). The reaction mixture was cooled to room temperature and then filtered through Celite^®^ with EtOAc washings. The filtrate was concentrated under reduced pressure to provide crude products and then the residue was purified through a silica gel column to give the corresponding **10a**–**c**.

Preparation of **10a**. From compound **9c** (131 mg, 1.05 mmol), as that described in procedure 1, gave pure **10a** (298 mg, 96%); off-white solid; M.p.: 209.2–210.2 °C; ^1^H-NMR (400 MHz, DMSO-*d*_6_) δ 9.02 (s, 1H), 8.51 (s, 1H), 7.80 (s, 1H), 7.42 (d, *J* = 7.1 Hz, 1H), 6.99 (d, *J* = 2.1 Hz, 2H), 6.93–6.86 (m, 1H), 6.75 (d, *J* = 6.9 Hz, 1H), 6.61 (dd, *J* = 7.0, 5.0 Hz, 2H), 4.88 (s, 2H), 3.83 (s, 6H); ^13^C-NMR (101 MHz, DMSO-*d*_6_) δ 162.3, 161.0, 159.4, 152.8, 142.3, 134.6, 125.3, 124.8, 120.7, 119.6, 116.2, 115.6, 104.4, 101.7, 55.5; ESI-MS: *m*/*z* 379.1 [M + H]^+^.

Preparation of **10b.** From compound **9b** (131 mg, 1.05 mmol), as that described in procedure 1, gave pure **10b** (275 mg, 88%); yellow solid; M.p.: 180.0–181.2 °C; ^1^H-NMR (400 MHz, DMSO-*d*_6_) δ 8.96 (s, 1H), 8.28 (s, 1H), 7.76 (s, 1H), 6.95 (s, 2H), 6.88 (t, *J* = 7.6 Hz, 1H), 6.60 (s, 2H), 6.46 (d, *J* = 7.3 Hz, 1H), 4.71 (s, 2H), 3.81 (s, 6H), 2.06 (s, 3H); ^13^C-NMR (101 MHz, DMSO-*d*_6_) δ 162.6, 160.9, 160.1, 152.9, 152.5, 145.6, 136.5, 134.7, 126.4, 123.4, 120.3, 119.7, 117.8, 112.8, 104.4, 101.6, 55.5, 18.3; ESI-MS: *m*/*z* 393.2 [M + H]^+^.

Preparation of **10c**. From compound **9b** (131 mg, 1.05 mmol), as that described in procedure 1, gave pure **10c** (282 mg, 90%); yellow solid; M.p.: 192.0–193.2 °C; ^1^H-NMR (400 MHz, CDCl_3_) δ 8.80 (s, 1H), 7.40 (s, 1H), 7.28 (s, 1H), 6.84 (d, *J* = 1.8 Hz, 2H), 6.82 (s, 1H), 6.68 (s, 1H), 6.65 (d, *J* = 8.0 Hz, 1H), 6.53 (s, 1H), 3.85 (s, 7H), 2.30 (s, 3H); ^13^C-NMR (101 MHz, CDCl_3_) δ 163.0, 161.2, 159.8, 154.5, 152.4, 141.9, 136.8, 135.1, 126.3, 123.2, 122.2, 120.0, 119.2, 117.7, 105.0, 101.7, 55.5, 21.2; ESI-MS: *m*/*z* 393.2 [M + H]^+^.

#### 3.2.9. General Procedure 2 of the Synthesis of Compounds **11a**–**c**

Corresponding **10a**–**c** (0.35 mmol) was dissolved in anhydrous THF or anhydrous DCM (5 mL), and sulfonyl chloride (290 mg, 2.1 mmol) was added to the solution in portions at −10 °C. The resulting mixture stirred at 0 °C for 1 h (monitored by TLC). EA (20 mL) and Na_2_CO_3_ solution (20 mL) were added, and the layers were partitioned and separated. The organic layers were washed with water and brine, dried over anhydrous sodium sulfate, filtered and then the filtration was concentrated in the vacuum to give corresponding **11a**–**c**.

Preparation of **11a**. From compound **10a** (133 mg, 0.35 mmol), as that described in procedure 2, gave pure **11a** (128 mg, 82%); yellow solid; M.p.: >250 °C; ^1^H-NMR (400 MHz, CDCl_3_) δ 8.89 (s, 1H), 7.55–7.39 (m, 1H), 7.13 (s, 1H), 7.07 (m, 1H), 6.92–6.81 (m, 2H), 6.74 (s, 1H), 6.68 (s, 1H), 3.97 (s, 6H), 3.93–3.78 (m, 2H); ^13^C-NMR (101 MHz, CDCl_3_) δ 162.2, 159.3, 154.7, 152.7, 148.4, 141.4, 133.4, 126.5, 126.1, 125.6, 124.7, 123.7, 119.4, 117.2, 115.3, 98.2, 56.7; ESI-MS: *m*/*z* 447.0 [M + H]^+^.

Preparation of **11b**. From compound **10b** (138 mg, 0.35 mmol), as that described in procedure 2, gave pure **11b** (127 mg, 79%); yellow solid; M.p.: >250 °C; ^1^H-NMR (400 MHz, DMSO-*d*_6_) δ 9.02 (s, 1H), 8.32 (s, 1H), 7.14 (s, 1H), 7.07 (s, 1H), 6.87 (t, *J* = 7.7 Hz, 1H), 6.59 (d, *J* = 7.8 Hz, 1H), 6.46 (d, *J* = 7.3 Hz, 1H), 4.72 (s, 2H), 3.97 (s, 6H), 2.07 (s, 3H); ^13^C-NMR (101 MHz, DMSO-*d*_6_) δ 161.8, 160.1, 154.5, 153.2, 145.7, 136.5, 132.6, 126.5, 124.2, 123.3, 121.5, 117.7, 113.4, 112.8, 99.4, 56.9, 18.3; ESI-MS: *m*/*z* 461.0 [M + H]^+^.

Preparation of **11c**. From compound **10c** (138 mg, 0.35 mmol), as that described in procedure 2, gave pure **11c** (131 mg, 81%); yellow solid; M.p.: 229.6–231.4 °C; ^1^H-NMR (400 MHz, CDCl_3_) δ 8.87 (s, 1H), 7.27 (m, 1H), 7.11 (s, 1H), 6.80 (s, 1H), 6.67 (s, 2H), 3.97 (s, 6H), 2.29 (s, 3H); ^13^C-NMR (101 MHz, CDCl_3_) δ 162.3, 159.7, 154.7, 152.7, 148.3, 141.9, 136.8, 133.4, 126.3, 124.7, 123.4, 123.2, 120.0, 117.7, 115.3, 98.2, 56.1, 21.2; ESI-MS: *m*/*z* 461.0 [M + H]^+^.

#### 3.2.10. General Procedure 3 of the Synthesis of Compounds **12**–**17**

One of corresponding **10a**–**c** and **11a**–**c** (0.1 mmol) was dissolved in anhydrous DCM. DIPEA (0.04 mL) and 0.1 mL 10% Acryloyl chloride (anhydrous THF) were added to the solution dropwise at −10 °C. The resulting mixture was stirred for 1 h (monitored by TLC), DCM (20 mL) and NaHCO_3_ solution (20 mL) were then added, and the layers were partitioned and separated. The organic layers were washed with NH_4_Cl solution, washed with water and brine, dried over anhydrous sodium sulfate, filtered and the solution was then concentrated under reduced pressure to give corresponding crude product **12**–**17**, which was furthermore purified through a silica gel column.

Preparation of **12**. From compound **10a** (38 mg, 0.1 mmol), as that described in procedure 3, gave pure **12** (21 mg, 50%); yellow solid; M.p.: 217.1–218.0 °C; ^1^H-NMR (400 MHz, DMSO-*d*_6_) δ 9.88 (s, 1H), 9.08 (s, 1H), 8.64 (s, 1H), 7.92 (d, *J* = 7.9 Hz, 1H), 7.84 (s, 1H), 7.56 (d, *J* = 8.2 Hz, 1H), 7.23 (t, *J* = 7.6 Hz, 1H), 7.12 (t, *J* = 7.6 Hz, 1H), 7.00 (s, 2H), 6.63 (s, 1H), 6.51 (dd, *J* = 16.6, 10.2 Hz, 1H), 6.28 (d, *J* = 17.0 Hz, 1H), 5.77 (d, *J* = 10.5 Hz, 1H), 3.83 (s, 6H); ^13^C-NMR (101 MHz, DMSO-*d*_6_) δ 163.8, 162.1, 161.0, 158.4, 153.4, 153.0, 134.5, 132.8, 131.5, 129.7, 127.2, 125.3, 124.6, 124.0, 123.4, 121.8, 119.5, 104.5, 101.8, 55.5; HPLC: method 1, room temperature, t_g_ = 11.72 min, UV_254_ = 98%; HRMS (ESI) *m*/*z* calcd for C_23_H_20_N_4_O_3_S [M + H]^+^: 433.1329, found: 433.1350.

Preparation of **13**. From compound **10b** (39 mg, 0.1 mmol), as that described in procedure 3, gave pure **13** (18 mg, 40%); yellow solid; M.p.:184.5–185.6 °C; ^1^H-NMR (400 MHz, DMSO-*d*_6_) δ 9.52 (s, 1H), 8.99 (s, 1H), 8.33 (s, 1H), 7.77 (s, 1H), 7.71 (d, *J* = 7.0 Hz, 1H), 7.18 (t, *J* = 7.9 Hz, 1H), 7.10 (d, *J* = 7.6 Hz, 1H), 6.96 (d, *J* = 1.8 Hz, 2H), 6.60 (s, 1H), 6.51 (dd, *J* = 16.8, 10.1 Hz, 1H), 6.21 (d, *J* = 16.7 Hz, 1H), 5.69 (d, *J* = 10.3 Hz, 1H), 3.81 (s, 6H), 2.16 (s, 3H); ^13^C-NMR (101 MHz, DMSO-*d*_6_) δ 168.8, 163. 5, 162.4, 160.9, 159.5, 152.9, 150.8, 136.9, 134.9, 134.6, 131.8, 126.8, 126.5, 125.9, 121.0, 120.0, 104.4, 101.7, 55.5, 18.5; HPLC: method 2, room temperature, t_g_ = 11.67 min, UV_254_ = 97%; HRMS (ESI) *m*/*z* calcd for C_24_H_22_N_4_O_3_S [M + H]^+^: 447.1485, found: 447.1495.

Preparation of **14**. From compound **10c** (39 mg, 0.1 mmol), as that described in procedure 3, gave pure **14** (16 mg, 36%); light yellow solid; M.p.: 239.0–240.1 °C; ^1^H-NMR (400 MHz, DMSO-*d*_6_) δ 9.82 (s, 1H), 9.05 (s, 1H), 8.55 (s, 1H), 7.82 (s, 1H), 7.74 (d, *J* = 8.2 Hz, 1H), 7.40 (s, 1H), 7.04 (d, *J* = 8.3 Hz, 1H), 6.99 (d, *J* = 2.1 Hz, 2H), 6.62 (s, 1H), 6.50 (dd, *J* = 17.0, 10.2 Hz, 1H), 6.28 (d, *J* = 17.0 Hz, 1H), 5.76 (t, *J* = 5.0 Hz, 1H), 3.83 (s, 6H), 3.34 (s, 4H), 2.50 (s, 4H), 2.31 (s, 3H); ^13^C-NMR (101 MHz, DMSO-*d*_6_) δ 163.6, 162.2, 161.0, 158.6, 153.2, 153.0, 134.5, 132.7, 131.5, 130.1, 129.8, 127.1, 125.8, 124.7, 124.3, 121.5, 119.6, 104.5, 101.8, 55.5, 20.5; HPLC: method 2, room temperature, t_g_ = 12.05 min, UV_254_ = 98%; HRMS (ESI) *m*/*z* calcd for C_24_H_22_N_4_O_3_S [M+Na]^+^: 469.1305, found: 469.1299.

Preparation of **15**. From compound **11a** (45 mg, 0.1 mmol), as that described in procedure 3, gave pure **15** (22 mg, 44%); white solid; M.p.: 236.6–236.9 °C; ^1^H-NMR (400 MHz, CDCl_3_) δ 8.91 (s, 1H), 8.63 (s, 1H), 7.93 (s, 1H), 7.52 (s, 1H), 7.38 (s, 1H), 7.22 (d, *J* = 8.2 Hz, 1H), 7.15 (s, 1H), 6.68 (s, 1H), 6.35 (d, *J* = 16.9 Hz, 1H), 6.20 (dd, *J* = 16.8, 10.3 Hz, 1H), 5.69 (d, *J* = 10.2 Hz, 1H), 3.98 (s, 6H); ^13^C-NMR (101 MHz, DMSO-*d*_6_) δ 163.7, 161.2, 158.3, 154.5, 153.3, 148.0, 132.6, 132.4, 131.5, 130.1, 127.1, 125.3, 124.5, 124.4, 124.2, 123.7, 123.0, 113.3, 99.5, 56.9; HPLC: method 2, room temperature, t_g_ = 11.37 min, UV_254_ = 97%; HRMS (ESI) *m*/*z* calcd for C_23_H_18_Cl_2_N_4_O_3_S [M+Na]^+^: 523.0369, found: 523.0371.

Preparation of **16**. From compound **11b** (46 mg, 0.1 mmol), as that described in procedure 3, gave pure **16** (24 mg, 47%); light yellow solid; M.p.: 142.5–143.8 °C; ^1^H-NMR (400 MHz, CDCl_3_) δ 8.89 (s, 1H), 8.49 (s, 1H), 8.05 (s, 1H), 7.24 (m, 1H), 7.13 (s, 1H), 7.09 (d, *J* = 7.5 Hz, 1H), 6.68 (s, 1H), 6.60 (s, 1H), 6.30 (d, *J* = 17.0 Hz, 1H), 6.14 (dd, *J* = 16.8, 10.2 Hz, 1H), 5.65 (d, *J* = 10.2 Hz, 1H), 3.98 (s, 6H), 2.31 (s, 3H); ^13^C-NMR (101 MHz, DMSO-*d*_6_) δ 163.5, 161.6, 159.5, 154.5, 153.2, 147.4, 136.9, 135.0, 132.5, 131.9, 126.8, 126.4, 125.9, 124.2, 122.2, 120.8, 113.4, 99.4, 56.9, 18.5; HPLC: method 2, room temperature, t_g_ = 11.12 min, UV_254_ = 98%; HRMS (ESI) *m*/*z* calcd for C_24_H_20_Cl_2_N_4_O_3_S [M + H]^+^: 515.0706, found: 515.0715.

Preparation of **17**. From compound **11c** (46 mg, 0.1 mmol), as that described in Procedure 3, gave pure **17** (22 mg, 44%); white solid; M.p.: 258.2–259.6 °C; ^1^H-NMR (400 MHz, DMSO-*d*_6_) δ 9.81 (s, 1H), 9.11 (s, 1H), 8.59 (s, 1H), 7.69 (d, *J* = 8.2 Hz, 1H), 7.41 (s, 1H), 7.23 (s, 1H), 7.08 (s, 1H), 7.03 (d, *J* = 8.3 Hz, 1H), 6.50 (dd, *J* = 17.0, 10.2 Hz, 1H), 6.27 (dd, *J* = 17.0, 1.7 Hz, 1H), 5.76 (dd, *J* = 10.1, 1.7 Hz, 1H), 3.98 (s, 6H), 2.30 (s, 3H); ^13^C-NMR (101 MHz, DMSO-*d*_6_) δ 163.6, 161.3, 158.6, 154.5, 153.3, 147.7, 133.0, 132.4, 131.5, 130.2, 129.9, 127.0, 125.8, 124.7, 124.6, 124.2, 122.7, 113.3, 99.4, 56.9, 20.5; HPLC: method 2, room temperature, t_g_ = 11.54 min, UV_254_ = 98%; HRMS (ESI) *m*/*z* calcd for C_24_H_20_Cl_2_N_4_O_3_S [M + H]^+^: 515.0706, found: 515.0723.

#### 3.2.11. General Procedure 4 of the Synthesis of Compounds **18**–**29**

Step 1. One of corresponding **10a**–**c** and **11a**–**c** (0.2 mmol) was dissolved in anhydrous DCM. DIPEA (0.07 mL) and (*E*)-4-bromobut-2-enoyl chloride (0.01 g/mL in anhydrous THF, 3 mL) were added to the solution at −5 °C. The resulting mixture was stirred for 2 h (monitored by TLC), DCM (10 mL) and water (10 mL) were added, the layers were partitioned and separated. The organic layers were washed with NaHCO_3_ solution, water and brine, dried over anhydrous sodium sulfate, filtered and the solution was concentrated under reduced pressure, used directly in the next step.

Step 2. One of corresponding crude product in the Step 1 (0.1 mmol) was dissolved in anhydrous DMF. NaI (46 mg, 0.3 mmol) and 0.07 mL (40%) dimethylamine solution in water were added. The resulting mixture was stirred at room temperature (monitored by TLC). EA (10 mL) and water (10 mL) were added, and the layers were partitioned and separated. The organic layers were washed with water and brine, dried over anhydrous sodium sulfate, filtered and concentrated, and then the residue was purified through a silica gel column to give corresponding **18**–**23**.

Preparation of **18**. From compound **10a** (38 mg, 0.1 mmol), as that described in step 1 and step 2 of procedure 4, gave pure **18** (24 mg, 49%); yellow solid; M.p.: 175.1–175.9 °C; ^1^H-NMR (400 MHz, CDCl_3_) δ 8.81 (s, 1H), 8.55 (s, 1H), 7.88 (s, 1H), 7.58 (s, 2H), 7.40 (s, 1H), 7.22 (d, *J* = 4.1 Hz, 2H), 6.92 (dt, *J* = 12.2, 5.9 Hz, 1H), 6.84 (d, *J* = 2.1 Hz, 2H), 6.53 (t, *J* = 2.1 Hz, 1H), 6.06 (d, *J* = 15.4 Hz, 1H), 3.85 (s, 6H), 3.05 (d, *J* = 5.7 Hz, 2H), 2.22 (s, 6H); ^13^C-NMR (101 MHz, CDCl_3_) δ 172.5, 164.0, 162.6, 161.3, 159.1, 155.3, 152.3, 141.8, 134.9, 131.7, 126.2, 125.6, 124.9, 124.6, 122.9, 118.9, 105.0, 101.9, 60.2, 55.6, 45.3; HPLC: method 1, room temperature, t_g_ = 11.94 min, UV_280_ = 96%; HRMS (ESI) *m*/*z* calcd for C_26_H_27_N_5_O_3_S [M + H]^+^: 490.1907, found: 490.1940.

Preparation of **19**. From compound **10b** (39 mg, 0.1 mmol), as that described in step 1 and step 2 of procedure 4, gave pure **19** (20 mg, 40%); yellow solid; M.p.: 151.2–152.9 °C; ^1^H-NMR (400 MHz, CDCl_3_) δ 8.79 (s, 1H), 8.37 (s, 1H), 8.08 (s, 1H), 7.36 (s, 1H), 7.24 (m, *J* = 8.0 Hz, 1H), 7.08 (d, *J* = 7.5 Hz, 1H), 6.91–6.84 (m, 2H), 6.83 (d, *J* = 2.2 Hz, 2H), 6.53 (t, *J* = 2.1 Hz, 1H), 5.99 (d, *J* = 15.3 Hz, 1H), 3.85 (s, 6H), 3.10–2.90 (m, 2H), 2.27 (s, 3H), 2.19 (s, 6H); ^13^C-NMR (101 MHz, CDCl_3_) δ 163.7, 162. 9, 161.3, 159.7, 155.4, 152.5, 141.7, 135.5, 135.2, 134.9, 127.2, 126.6, 126.3, 122.9, 120.8, 118.9, 105.0, 101.9, 60.2, 55.7, 45.4, 18.7; HPLC: method 1, room temperature, t_g_ = 11.87 min, UV_254_ = 98%; HRMS (ESI) *m*/*z* calcd for C_27_H_29_N_5_O_3_S [M + H]^+^: 504.2064, found: 504.2082.

Preparation of **20**. From compound **10c** (39 mg, 0.1 mmol), as that described in step 1 and step 2 of procedure 4, gave pure **20** (17 mg, 34%); yellow solid; M.p.: 170.8–171.5 °C; ^1^H-NMR (400 MHz, DMSO-*d*_6_) δ 9.78 (s, 1H), 9.06 (s, 1H), 8.56 (s, 1H), 7.82 (s, 1H), 7.73 (d, *J* = 8.2 Hz, 1H), 7.41 (s, 1H), 7.04 (d, *J* = 8.5 Hz, 1H), 7.00 (s, 2H), 6.84–6.71 (m, 1H), 6.64 (s, 1H), 6.39 (d, *J* = 15.9 Hz, 1H), 3.84 (s, 6H), 3.27 (s, 2H), 2.32 (s, 9H); ^13^C-NMR (101 MHz, DMSO-*d*_6_) δ 163.5, 162.2, 161.0, 158.6, 153.2, 153.0, 134.5, 132.7, 130.0, 126.3, 125.7, 124.6, 124.4, 121.5, 119.6, 104.5, 101.8, 59.3, 55.5, 44.7, 20.5; HPLC: method 1, room temperature, t_g_ = 12.46 min, UV_254_ = 99%; HRMS (ESI) *m*/*z* calcd for C_27_H_29_N_5_O_3_S [M + H]^+^: 504.2064, found: 504.2092.

Preparation of **21**. From compound **11a** (45 mg, 0.1 mmol), as that described in step 1 and step 2 of procedure 4, gave pure **21** (21 mg, 38%); yellow solid; M.p.: 231.7–232.5 °C; ^1^H-NMR (400 MHz, DMSO-*d*_6_) δ 9.79 (s, 1H), 9.14 (s, 1H), 8.70 (s, 1H), 7.87 (d, *J* = 7.9 Hz, 1H), 7.57 (d, *J* = 7.6 Hz, 1H), 7.26 (s, 1H), 7.21 (t, *J* = 7.5 Hz, 1H), 7.12 (d, *J* = 7.6 Hz, 1H), 7.08 (s, 1H), 6.76 (dt, *J* = 15.2, 5.8 Hz, 1H), 6.34 (d, *J* = 15.5 Hz, 1H), 3.98 (s, 6H), 3.05 (d, *J* = 5.5 Hz, 2H), 2.16 (s, 6H); ^13^C-NMR (101 MHz, DMSO-*d*_6_) δ 163.8, 161.3, 158.4, 154.5, 153.4, 147. 8, 141.8, 132.4, 130.2, 125.4, 125.1, 124.4, 124.2, 123.6, 123.0, 113.3, 99.5, 59.7, 56.9, 45.2; HPLC: method 1, room temperature, t_g_ = 14.20 min, UV_254_ = 98%; HRMS (ESI) *m*/*z* calcd for C_26_H_25_Cl_2_N_5_O_3_S [M + H]^+^: 558.1128, found: 558.1158.

Preparation of **22.** From compound **11b** (46 mg, 0.1 mmol), as that described in step 1 and step 2 of procedure 4, gave pure **22** (24 mg, 42%); yellow solid; M.p.: >250 °C; ^1^H-NMR (400 MHz, DMSO-*d*_6_) δ 9.50 (s, 1H), 9.04 (s, 1H), 8.40 (s, 1H), 7.74 (d, *J* = 7.7 Hz, 1H), 7.18 (t, *J* = 7.8 Hz, 2H), 7.08 (d, *J* = 8.2 Hz, 2H), 6.70 (dt, *J* = 15.3, 6.2 Hz, 1H), 6.41 (d, *J* = 15.4 Hz, 1H), 3.97 (s, 6H), 3.21 (d, *J* = 4.4 Hz, 2H), 2.28 (s, 6H), 2.16 (s, 3H); ^13^C-NMR (101 MHz, DMSO-*d*_6_) δ 163.2, 161.6, 159.5, 154.5, 153.2, 147.4, 136.9, 135.2, 132.5, 130.3, 126.3, 125.9, 124.2, 122.2, 120.6, 113.3, 99.4, 58.9, 56.9, 44.0, 18.5; HPLC: method 2, room temperature, t_g_ = 11.35 min, UV_254_ = 99%; HRMS (ESI) *m*/*z* calcd for C_27_H_27_Cl_2_N_5_O_3_S [M + H]^+^: 572.1284, found: 572.1327.

Preparation of **23**. From compound **11c** (46 mg, 0.1 mmol), as that described in step 1 and step 2 of procedure 4, gave pure **23** (19 mg, 33%); yellow solid; M.p.: 214.0–214.6 °C; ^1^H-NMR (400 MHz, CDCl_3_) δ 8.89 (s, 1H), 8.45 (s, 1H), 7.80 (s, 1H), 7.36 (d, *J* = 7.2 Hz, 1H), 7.16 (s, 1H), 7.14 (s, 1H), 7.00 (d, *J* = 7.8 Hz, 1H), 6.91 (dt, *J* = 15.0, 6.0 Hz, 1H), 6.68 (s, 1H), 6.03 (d, *J* = 15.4 Hz, 1H), 5.30 (s, 1H), 3.98 (s, 6H), 3.07 (d, *J* = 5.8 Hz, 2H), 2.36 (s, 3H), 2.24 (s, 6H); ^13^C-NMR (101 MHz, CDCl_3_) δ 163.8, 161.9, 159.3, 154.7, 152.8, 149.0, 141.5, 133.2, 126.4, 126.3, 125.2, 124.7, 124.4, 124.1 115.2, 98.2, 60.2, 56.7, 45.3, 21.2; HPLC: method 1, room temperature, t_g_ = 12.66 min, UV_254_ = 95%; HRMS (ESI) *m*/*z* calcd for C_27_H_27_Cl_2_N_5_O_3_S [M + Na]^+^: 594.1104, found: 594.1091.

Step 3. Corresponding crude product in the step 1 (0.1 mmol) was dissolved in anhydrous DMF. NaI (46 mg, 0.3 mmol) and 0.02 mL *N*-Methylpiperazine were added. The resulting mixture was stirred at room temperature (monitored by TLC). EA (10 mL) and water (10 mL) were added, and the layers were partitioned and separated. The organic layers were washed with water and brine, and dried over anhydrous sodium sulfate. Filtered and the solution concentrated under reduced pressure, and then the residue was purified through a silica gel column to give corresponding **24**–**29**.

Preparation of **24**. From compound **10a** (38 mg, 0.1 mmol), as that described in step 1 and step 3 of procedure 4, gave pure **24** (23 mg, 43%); yellow solid; M.p.: 182.9–183.7 °C; ^1^H-NMR (400 MHz, CDCl_3_) δ 8.82 (s, 1H), 8.50 (s, 1H), 7.88 (s, 1H), 7.55 (s, 1H), 7.48 (s, 1H), 7.41 (s, 1H), 7.21 (s, 2H), 6.91 (d, *J* = 9.3 Hz, 1H), 6.84 (d, *J* = 2.0 Hz, 2H), 6.53 (s, 1H), 6.04 (d, *J* = 15.3 Hz, 1H), 3.85 (s, 6H), 3.09 (d, *J* = 5.5 Hz, 2H), 2.42 (m, 8H), 2.23 (s, 3H); ^13^C-NMR (101 MHz, CDCl_3_) δ 163.9, 162.5, 161.3, 159.1, 155.4, 152.3, 141.7, 134.8, 131.7, 126.2, 125.7, 124.9, 124.7, 122.8, 118.8, 105.0, 101.8, 59.2, 55.6, 54.9, 53.2, 45.9; HPLC: method 1, room temperature, t_g_ = 11.04 min, UV_280_ = 96%; HRMS (ESI) *m*/*z* calcd for C_29_H_32_N_6_O_3_S [M+Na]^+^: 567.2149, found: 567.2169.

Preparation of **25**. From compound **10b** (39 mg, 0.1 mmol), as that described in step 1 and step 3 of procedure 4, gave pure **25** (20 mg, 36%); yellow solid; M.p.: 156.5–157.8 °C;^1^H-NMR (400 MHz, CDCl_3_) δ 8.83 (s, 1H), 8.58 (s, 1H), 8.00 (s, 1H), 7.39 (s, 1H), 7.23 (s, 1H), 7.10 (d, *J* = 7.0 Hz, 1H), 6.84 (s, 2H), 6.82–6.69 (m, 2H), 6.54 (s, 1H), 5.99 (d, *J* = 15.0 Hz, 1H), 3.86 (s, 6H), 3.32 (s, 2H), 3.16 (s, 2H), 2.83 (s, 6H), 2.68 (s, 3H), 2.29 (s, 4H); ^13^C-NMR (101 MHz, MeOD) δ 166.3, 164.0, 162.9, 160.7, 156.5, 153.9, 141.6, 138.4, 136.1, 135.9, 132.1, 128.9, 127.9, 127.7, 123.3, 119.7, 105.8, 102.7, 59.5, 56.1, 55.2, 52.4, 44.8, 18.8; HPLC: method 1, room temperature, t_g_ = 11.26 min, UV_280_ = 97%; HRMS (ESI) *m*/*z* calcd for C_30_H_34_N_6_O_3_S [M + H]^+^: 559.2486, found: 559.2494.

Preparation of **26**. From compound **10c** (39 mg, 0.1 mmol), as that described in step 1 and step 3 of procedure 4, gave pure **26** (19 mg, 34%); yellow solid; M.p.: 182.0–183.2 °C; ^1^H-NMR (400 MHz, CDCl_3_) δ 8.80 (s, 1H), 7.73 (s, 1H), 7.46 (d, *J* = 7.6 Hz, 1H), 7.37 (s, 1H), 7.02 (d, *J* = 7.3 Hz, 1H), 6.83 (d, *J* = 2.0 Hz, 2H), 6.82–6.72 (m, 1H), 6.53 (t, *J* = 1.9 Hz, 1H), 6.08 (d, *J* = 15.0 Hz, 1H), 3.85 (s, 6H), 3.17 (s, 2H), 3.14 (d, *J* = 5.5 Hz, 2H), 2.80 (s, 6H), 2.63 (s, 3H), 2.36 (s, 3H); ^13^C-NMR (101 MHz, CDCl_3_) δ 163.5, 161.3, 155.3, 139.8, 135.6, 134.8, 131.4, 129.0, 126.5, 125.0, 118.8, 105.0, 101.8, 58.1, 55.6, 53.8, 49.7, 43.5, 21.1; HPLC: method 1, room temperature, t_g_ = 11.87 min, UV_254_ = 99%; HRMS (ESI) *m*/*z* calcd for C_30_H_34_N_6_O_3_S [M+Na]^+^: 581.2305, found: 581.2278.

Preparation of **27**. From compound **11a** (45 mg, 0.1 mmol), as that described in step 1 and step 3 of procedure 4, gave pure **27** (21 mg, 34%); off-white solid; M.p.: 160.1–160.7 °C; ^1^H-NMR (400 MHz, CDCl_3_) δ 8.91 (s, 1H), 8.46 (s, 1H), 7.88 (s, 1H), 7.56 (s, 1H), 7.29 (s, 1H), 7.22 (s, 2H), 7.16 (s, 1H), 6.93 (dt, *J* = 15.1, 6.1 Hz, 1H), 6.68 (s, 1H), 6.05 (d, *J* = 15.4 Hz, 1H), 3.98 (s, 6H), 3.11 (d, *J* = 5.8 Hz, 2H), 2.45 (s, 8H), 2.27 (s, 3H); ^13^C-NMR (101 MHz, CDCl_3_) δ 163.9, 161.8, 159.0, 154.7, 152.7, 149.1, 141.9, 133.1, 131.6, 126.2, 125.7, 124.9, 124.7, 124.4, 124.2, 115.1, 98.2, 59.3, 56.7, 55.0, 53.3, 46.0; HPLC: method 1, room temperature, t_g_ = 11.63 min, UV_254_ = 97%; HRMS (ESI) *m*/*z* calcd for C_29_H_30_Cl_2_N_6_O_3_S [M + H]^+^: 613.1550, found: 613.1577.

Preparation of **28**. From compound **11b** (46 mg, 0.1 mmol), as that described in step 1 and step 3 of procedure 4, gave pure **28** (18 mg, 29%); white solid; M.p.: 225.0–226.2 °C; ^1^H-NMR (400 MHz, CDCl_3_) δ 8.85 (s, 1H), 8.46 (s, 1H), 8.01 (s, 1H), 7.23 (t, *J* = 7.9 Hz, 1H), 7.09 (s, 1H), 7.07 (d, *J* = 7.4 Hz, 1H), 6.96 (s, 1H), 6.87 (dt, *J* = 15.3, 6.2 Hz, 1H), 6.68 (s, 1H), 6.01 (d, *J* = 15.3 Hz, 1H), 3.97 (s, 6H), 3.07 (d, *J* = 6.1 Hz, 2H), 2.43 (s, 8H), 2.28 (s, 3H), 2.26 (s, 3H); ^13^C-NMR (101 MHz, CDCl_3_) δ 172.6, 163.7, 162.1, 159.5, 154.7, 152.9, 149.1, 141.5, 135.6, 135.1, 133.1, 127.1, 126.7, 124.4, 124.0, 115.2, 98.3, 59.2, 56.7, 55.0, 53.2, 45.9, 18.8; HPLC: method 1, room temperature, t_g_ = 11.50 min, UV_254_ = 99%; HRMS (ESI) *m*/*z* calcd for C_30_H_32_Cl_2_N_6_O_3_S [M + H]^+^: 627.1706, found: 627.1736.

Preparation of **29**. From compound **11c** (46 mg, 0.1 mmol), as that described in step 1 and step 3 of procedure 4, gave pure **29** (22 mg, 35%); white solid; M.p.: 212.4–212.9 °C; ^1^H-NMR (400 MHz, CDCl_3_) δ 8.82 (s, 1H), 8.39 (s, 1H), 7.69 (s, 1H), 7.30 (d, *J* = 6.6 Hz, 1H), 7.14 (d, *J* = 13.1 Hz, 1H), 7.07 (s, 1H), 6.93 (d, *J* = 7.6 Hz, 1H), 6.85 (dd, *J* = 13.8, 7.4 Hz, 1H), 6.61 (s, 1H), 5.95 (d, *J* = 15.3 Hz, 1H), 3.90 (s, 6H), 3.03 (d, *J* = 5.9 Hz, 2H), 2.40 (s, 8H), 2.29 (s, 3H), 2.22 (s, 3H); ^13^C-NMR (101 MHz, DMSO-*d*_6_) δ 163.7, 161.3, 158.6, 154.5, 153.3, 147.7, 141.1, 133.1, 132.4, 130.3, 125.8, 125.7, 124.7, 125.6, 124.2, 122.7, 113.3, 99.4, 58.3, 56.8, 54.4, 52.4, 45.4, 20.5; HPLC: method 1, room temperature, t_g_ = 12.03 min, UV_254_ = 96%; HRMS (ESI) *m*/*z* calcd for C_30_H_32_Cl_2_N_6_O_3_S [M + Na]^+^: 649.1526, found: 649.1482.

#### 3.3. General Procedure for In Vitro Cell-Proliferation Assays

NCI-H1975, Ramos, and SNU-16 cells were maintained in Revolutions-Per-minute Indicator 1640 (RPMI 1640) medium (Gibco, Grand Island, NY, USA), A431 was maintained in Dulbecco’s Modified Eagle Medium (DMEM) medium (Gibco), MDA-MB-231 was cultured in L-15 medium (Gibco), and NCI-H1581 was cultured in ACL-4 (Gibco), and all of them were supplemented with 10% heat-inactivated fetal calf serum (FBS; Gibco) at 37 °C in a 5% CO_2_ humidiﬁed environment. Cells in 96-well plates were treated with gradient concentrations of compounds at 37 °C for 72 h. Cell proliferation assays in NCI-H1975, A431, MDA-MB-231 and NCI-H1581 were determined by using sulforhodamine B (SRB; Sigma, St. Louis, MO, USA). The antiproliferative activity of compounds, examined in Ramos and SUN-16 cell lines, was determined by using CCK-8 (Dojindo Laboratories, Kamimashiki-gun, Japan). The dosages corresponding to the half-maximal inhibition (IC_50_) were calculated using SoftMax pro-based nonlinear 4-parameter regression analysis (*n* = 3).

## 4. Conclusions

In summary, a series of 2,6-substituted thieno[3,2-*d*]pyrimidine containing electrophilic warheads derivatives were designed and synthesized. The derivatives possessed moderate antiproliferative properties against four tested cancer cell lines (A431, NCI-H1975, Ramos and SNU-16). Compound **12** showed the most potent cytotoxic activity on the four cell lines above with IC_50_ values of 1.4 μM, 1.2 μM, 0.6 μM, and 2.6 μM, respectively. However, the antiproliferative activity of **12** against MDA-MB-221 indicated that **12** had the selectivity towards certain tumor cell lines. The preliminary investigation showed that the acrylamide electrophilic warhead was more active than the other two warheads. It was interesting to note that an *o*-Me substituent on the benzene ring attached to the electrophilic warhead weakens the activity slightly while *p*-Me substituent results in a significant loss of activity. Another interesting phenomenon was that a dramatic loss in antitumor activity was observed when **12**–**14** were transformed into their dichloride substituted derivatives **15**–**17**. In addition, compounds **12**–**29** demonstrated less than 40% inhibition against BTK at 500 nM, indicating that BTK might not be the biological target for these compounds. The enzymatic screen assays suggested that the selected twenty kinases might also not be the biological targets for the synthesized compounds. Thus, future efforts will focus on investigating the molecular mechanisms of the target compounds, especially **12**. In summary, compound **12** is worth further research as a new potential anticancer agent.

## Figures and Tables

**Table 1:**
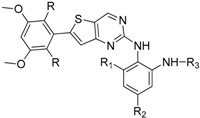
Structures of compounds **12**–**29**.

Compounds	R	R_1_	R_2_	R_3_	Compounds	R	R_1_	R_2_	R_3_
**12**	H	H	H	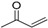	**21**	Cl	H	H	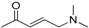
**13**	H	Me	H	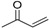	**22**	Cl	Me	H	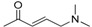
**14**	H	H	Me	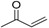	**23**	Cl	H	Me	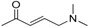
**15**	Cl	H	H	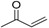	**24**	H	H	H	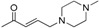
**16**	Cl	Me	H	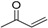	**25**	H	Me	H	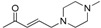
**17**	Cl	H	Me	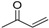	**26**	H	H	Me	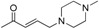
**18**	H	H	H	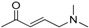	**27**	Cl	H	H	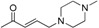
**19**	H	Me	H	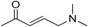	**28**	Cl	Me	H	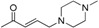
**20**	H	H	Me	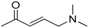	**29**	Cl	H	Me	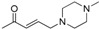

**Table 2:** Antiproliferative activity of **12**–**29** against A431, NCI-H1975, Ramos, and SNU-16.

Compounds	IC_50_^a^ ± SD (μM)			
	A431	NCI-H1975	Ramos	SNU-16
**12**	1.4 ± 0.2	1.2 ± 0.1	0.6 ± 0.3	2.6 ± 0.2
**13**	4.1 ± 0.1	5.4 ± 0.5	3.9 ± 0.8	6.7 ± 2.2
**14**	2.0 ± 0.2	2.8 ± 0.2	2.7 ± 0.8	3.2 ± 0.4
**15**	16.3 ± 1.6	12.6 ± 4.0	8.0 ± 2.4	19.2 ± 0.6
**16**	>20	>20	14.1 ± 4.4	>20
**17**	10.3 ± 1.8	9.7 ± 0.2	6.4 ± 3.5	14.2 ± 1.8
**18**	5.3 ± 0.4	12.2 ± 0.6	6.4 ± 1.9	11.6 ± 1.2
**19**	5.9 ± 0.4	9.4 ± 0.5	9.9 ± 1.2	16.3 ± 0.5
**20**	4.7 ± 0.1	5.0 ± 1.3	3.8 ± 0.4	6.2 ± 2.0
**21**	4.4 ± 0.2	7.2 ± 0.5	4.6 ± 1.9	9.1 ± 0.4
**22**	7.1 ± 3.6	14.0 ± 3.7	7.6 ± 2.2	>20
**23**	4.5 ± 0.2	6.8 ± 0.04	7.5 ± 1.1	6.1 ± 0.9
**24**	10.5 ± 0.2	14.3 ± 3.1	7.0 ± 1.9	>20
**25**	>20	>20	>20	>20
**26**	>20	>20	17.3 ± 4.1	>20
**27**	12.2 ± 1.1	12.1 ± 2.3	11.9 ± 2.8	14.8 ± 0.6
**28**	10.9 ± 4.0	11.2 ± 0.6	12.8 ± 4.3	>20
**29**	11.2 ± 681.1	11.3 ± 1.3	10.2 ± 0.1	12.2 ± 2.7
**ADR ^b^**	0.2 ± 0.05	0.3 ± 0.03	0.3 ± 0.01	0.3 ± 0.05

^a^ Data presented is the mean ± SD value of three independent determinations; ^b^ Used as positive control.

**Table 3:** Antiproliferative activity of **12** against NCI-H1581, MDA-MB-231.

Compounds	IC_50_^a^ ± SD (μM)	
	NCI-H1581	MDA-MB-231
**12**	1.32 ± 0.08	>20
**ADR ^b^**	0.5 ± 0.03	0.4 ± 0.05

^a^ Data presented is the mean ± SD value of three independent determinations; ^b^ Used as positive control.

## Supplementary Materials

The following are available online. The general HPLC method, ESI-TOF-MS spectrums of **12**–**29**, FGFR1 and FGFR4 enzymatic assay, BTK enzymatic assay, and in vitro enzymatic assays, Figure S1. FGFR1 inhibition rate of **12**–**29** at the concentration of 10 nM, 100 nM and 1000 nM, Table S1: Positive controls in FGFR1 enzymatic assay, Figure S2. FGFR4 inhibition rate of **12**–**29** at the concentration of 10 nM, 100 nM and 1000 nM, Table S2: Positive controls in FGFR4 enzymatic assay, Figure S3. BTK inhibition rate of **12**–**29** at the concentration of 10 nM, 100 nM and 1000 nM, Table S3–S6: Some positive controls in in vitro enzymatic screen assays.

## Abbreviations

The following abbreviations are used in this manuscript:

FDA	Food and Drug Administration
EGFR	Epidermal growth factor receptor
TKI	Tyrosine kinase inhibitors
BTK	Brutons tyrosine kinase
JAK	Janus kinase
PI3K	Phosphatidylinositol 3 kinase
FGFR4	Fibroblast growth factor receptor-4
FGF19	Fibroblast growth factor 19
EA	Ethyl acetate
DMF	*N*,*N*-Dimethylformamide
DIPEA	*N*,*N*-Diisopropylethylamine
ADR	Doxorubicin
DMSO	Dimethyl sulfoxide
HPLC	High Performance Liquid Chromatography
TLC	Thin Layer Chromatography
PE	Petroleum ether
ESI MS	Electrospray ionization mass spectrometry
HRMS	High resolution mass
TMS	Tetramethylsilane
RPMI 1640	Revolutions-Per-minute Indicator 1640
DMEM	Dulbecco’s Modified Eagle Medium
ELISA	Enzyme-linked immunosorbent assay
