# Supplementary material for: Design, Synthesis and Anti-Proliferative Activities of 2,6-Substituted Thieno[3,2-d]pyrimidine Derivatives Containing Electrophilic Warheads
Source: Molecules. 2017 May 12;22(5):788. doi: 10.3390/molecules22050788 (PMC6154568; doi:10.3390/molecules22050788)
Supplement: Supplementary file 1 [file molecules-22-00788-s001.pdf]

Supplementary Data

Design, synthesis and anti-proliferative activities of 2,6-substituted thieno[3,2-*d*]pyrimidine derivatives containing electrophilic warheads

Qiumeng Zhang [1], Zonglong Hu [2], Qianqian Shen [2], Yi Chen [2,*] and Wei Lu [1,*]

[1] School of Chemistry and Molecular Engineering, East China Normal University, 3663 North Zhongshan Road, Shanghai 200062, P. R. China; qiumeng-zhang@foxmail.com (Q.Z.)

[2] Division of Anti-Tumor Pharmacology, State Key Laboratory of Drug Research, Shanghai Institute of Materia Medica, Chinese Academy of Sciences, Shanghai 201203, P. R. China; zlhu@jding.dhs.org (Z.H.); shenqianqian@simm.ac.cn (Q.S.)

* Correspondence: wlu@chem.ecnu.edu.cn; Tel.: +86-021-62238771 (W.L.);
ychen@simm.ac.cn; Tel.: +86-021-50801552 (Y.C.)

Table of contents	S1
General HPLC method	S2
ESI-TOF-MS spectrums of **12-29**	S3
FGFR1 and FGFR4 enzymatic assay	S4
BTK enzymatic assay	S5
In vitro enzymatic assays	S6

General HPLC Method

HPLC analysis was performed on an Agilent Technologies 1200 series using an Agilent Eclipse XDBC18 (250 mm × 4.6 mm).

Method 1: a mobile phase gradient from 5% MeCN/H_2O (1‰ TFA) to 95% MeCN/H2O (1‰ TFA) for 15 min and 95% MeCN/H_2O (1‰ TFA) for 3 min more, a flow rate of 1.0 mL/min.

Method 2: a mobile phase gradient from 5% MeOH/H_2O (90% H_2O and 10% MeOH) to 95% MeOH/H_2O (90% H_2O and 10% MeOH) for 8 min and 95% MeOH/H2O (90% H_2O and 10% MeOH) for 8 min more, a flow rate of 1.0 mL/min. The two methods were used to determine the purity for the tested compounds.

ESI-TOF-MS spectrums of 12-29

ESI-TOF-MS spectra of **12**

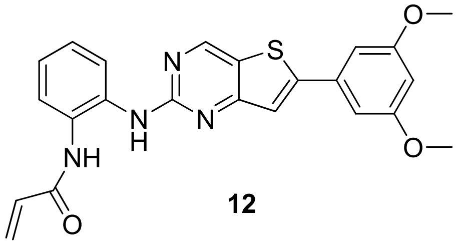

12

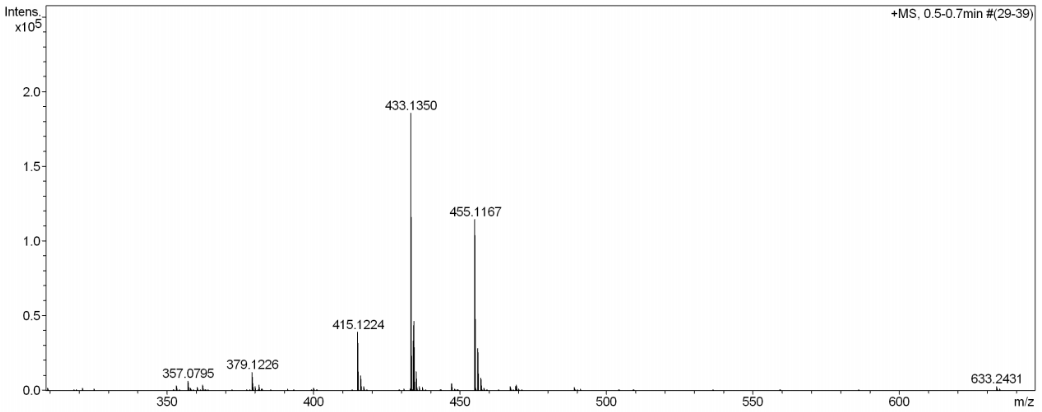

Meas. m/z	#	Formula	m/z	err [ppm]	Mean err [ppm]	rdb	N-Rule	e⁻ Conf	mSigma	Std I	Std Mean m/z	Std I VarNorm	Std m/z Diff	Std Comb Dev
433.1350														
	1	C 23 H 21 N 4 O 3 S	433.1329	-4.9	-4.3	15.5	ok	even	14.45	0.0278	0.0022	0.0097	0.0030	0.7085

ESI-TOF-MS spectra of **13**

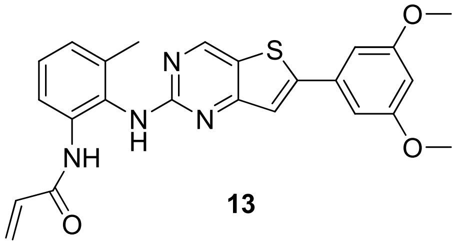

13

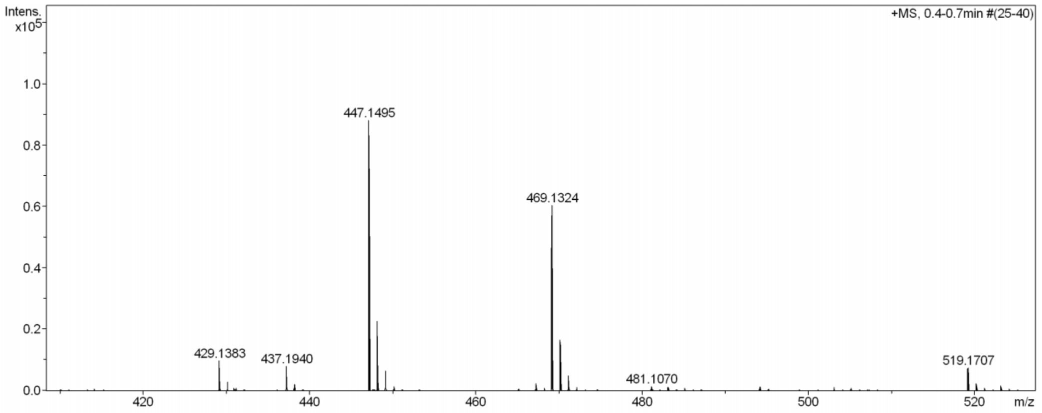

Meas. m/z	#	Formula	m/z	err [ppm]	Mean err [ppm]	rdb	N-Rule	e⁻ Conf	mSigma	Std I	Std Mean m/z	Std I VarNorm	Std m/z Diff	Std Comb Dev
447.1495														
	1	C 24 H 23 N 4 O 3 S	447.1485	-2.1	-2.3	15.5	ok	even	15.30	0.0274	0.0013	0.0093	0.0024	0.6671

ESI-TOF-MS spectra of **14**

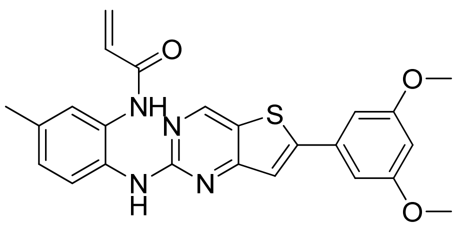

14

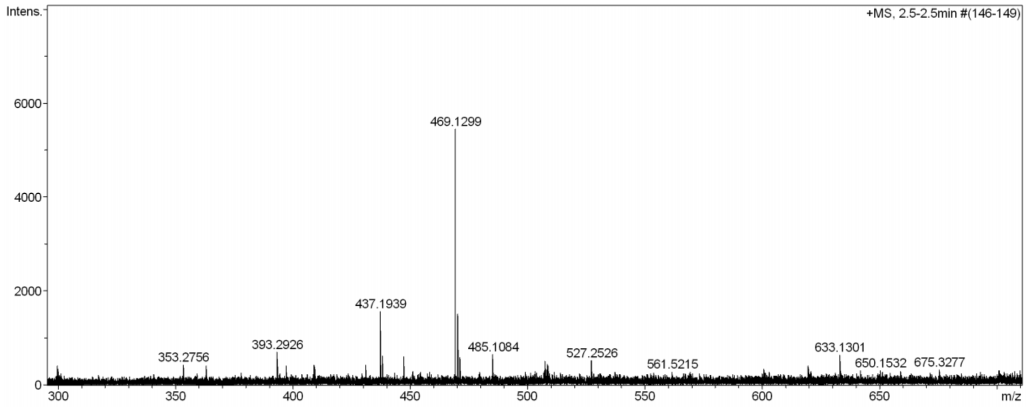

Meas. m/z	#	Formula	m/z	err [ppm]	Mean err [ppm]	rdb	N-Rule	e⁻ Conf	mSigma	Std I	Std Mean m/z	Std I VarNorm	Std m/z Diff	Std Comb Dev
469.1299														
	1	C 24 H 22 N 4 Na O 3 S	469.1305	1.2	0.6	15.5	ok	even	13.93	0.0269	0.0019	0.0128	0.0017	0.6423

ESI-TOF-MS spectra of **15**

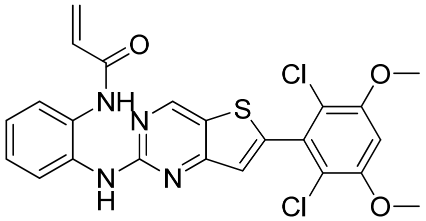

15

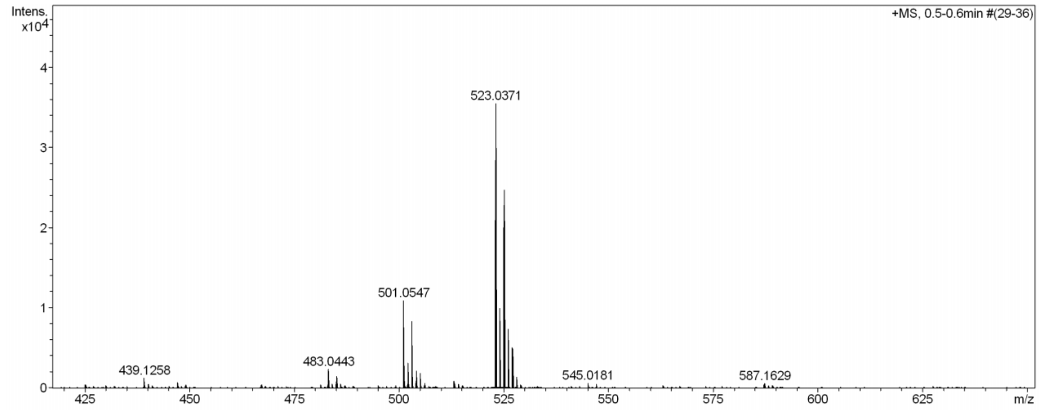

Meas. m/z	#	Formula	m/z	err [ppm]	Mean err [ppm]	rdb	N-Rule	e⁻ Conf	mSigma	Std I	Std Mean m/z	Std I VarNorm	Std m/z Diff	Std Comb Dev
523.0371														
	1	C 23 H 18 Cl 2 N 4 Na O 3 S	523.0369	-0.5	-0.6	15.5	ok	even	15.31	0.0161	0.0006	0.0047	0.0008	0.4824

ESI-TOF-MS spectra of **16**

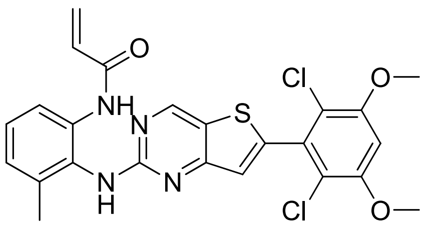

16

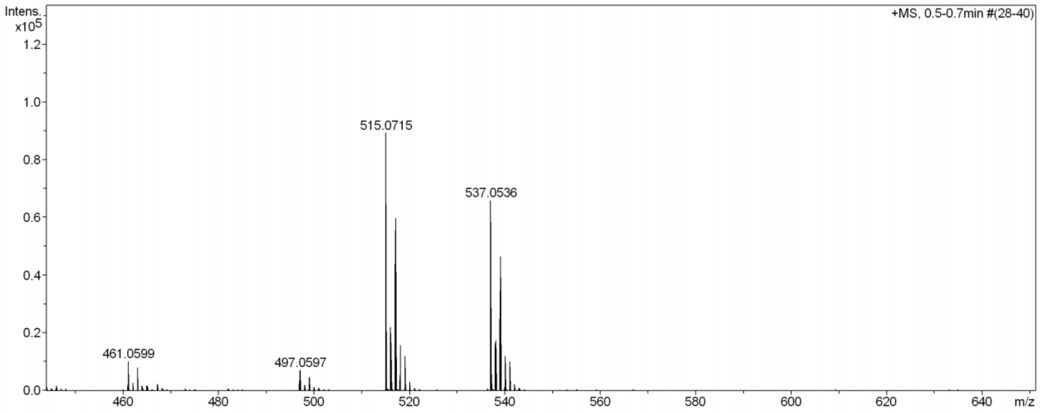

Meas. m/z	#	Formula	m/z	err [ppm]	Mean err [ppm]	rdb	N-Rule	e⁻ Conf	mSigma	Std I	Std Mean m/z	Std I VarNorm	Std m/z Diff	Std Comb Dev
515.0715														
	1	C 24 H 21 Cl 2 N 4 O 3 S	515.0706	-1.8	-1.3	15.5	ok	even	29.53	0.0304	0.0008	0.0073	0.0006	0.6482

ESI-TOF-MS spectra of **17**

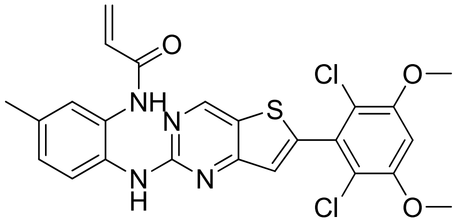

17

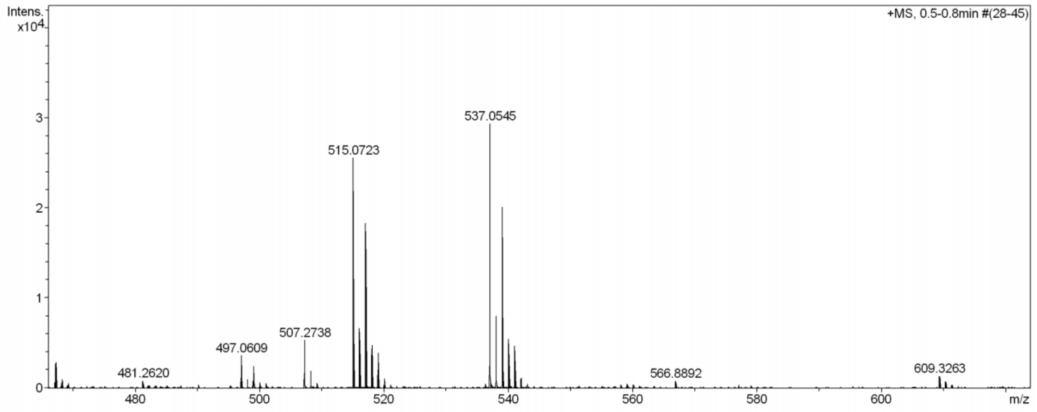

Meas. m/z	#	Formula	m/z	err [ppm]	Mean err [ppm]	rdb	N-Rule	e⁻ Conf	mSigma	Std I	Std Mean m/z	Std I VarNorm	Std m/z Diff	Std Comb Dev
515.0723														
	1	C 24 H 21 Cl 2 N 4 O 3 S	515.0706	-3.3	-2.8	15.5	ok	even	11.45	0.0122	0.0015	0.0033	0.0007	0.5891

ESI-TOF-MS spectra of **18**

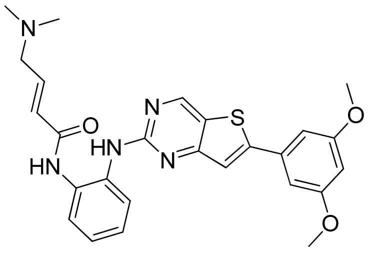

18

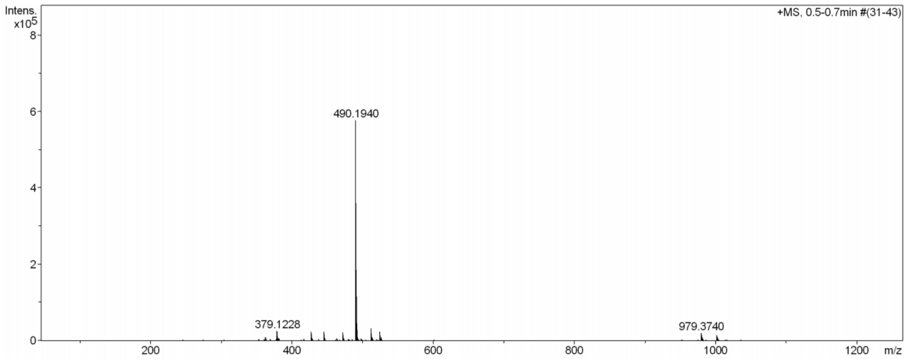

Meas. m/z	#	Formula	m/z	err [ppm]	Mean err [ppm]	rdb	N-Rule	e⁻ Conf	mSigma	Std I	Std Mean m/z	Std I VarNorm	Std m/z Diff	Std Comb Dev
490.1940														
	1	C 26 H 28 N 5 O 3 S	490.1907	-6.6	-6.1	15.5	ok	even	11.02	0.0162	0.0032	0.0073	0.0027	0.6881

ESI-TOF-MS spectra of **19**

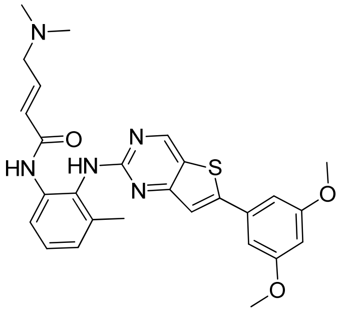

19

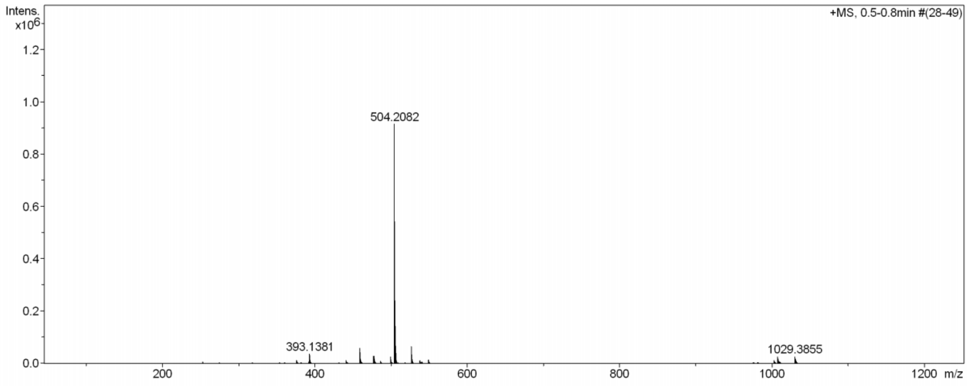

Meas. m/z	#	Formula	m/z	err [ppm]	Mean err [ppm]	rdb	N-Rule	e⁻ Conf	mSigma	Std I	Std Mean m/z	Std I VarNorm	Std m/z Diff	Std Comb Dev
504.2082														
	1	C 27 H 30 N 5 O 3 S	504.2064	-3.6	-3.7	15.5	ok	even	30.54	0.0547	0.0021	0.0179	0.0028	0.8191

ESI-TOF-MS spectra of **20**

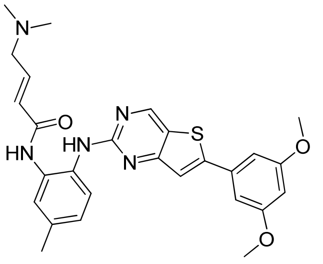

20

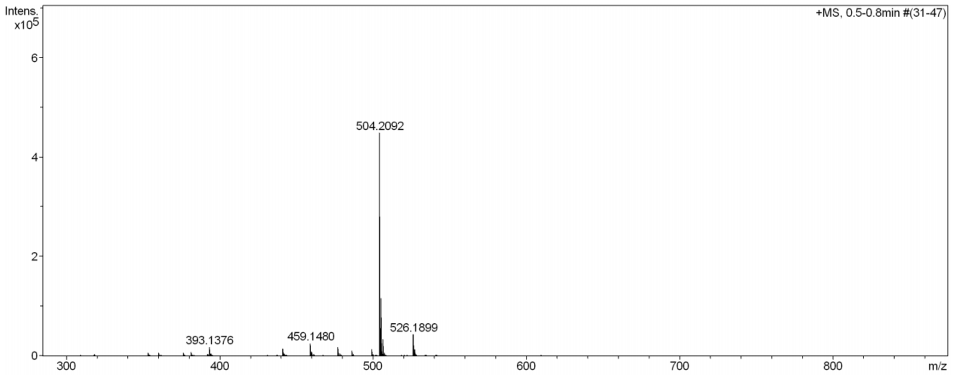

Meas. m/z	#	Formula	m/z	err [ppm]	Mean err [ppm]	rdb	N-Rule	e⁻ Conf	mSigma	Std I	Std Mean m/z	Std I VarNorm	Std m/z Diff	Std Comb Dev
504.2092	1	C 27 H 30 N 5 O 3 S	504.2064	-5.6	-5.3	15.5	ok	even	33.80	0.0595	0.0028	0.0193	0.0025	0.8634

ESI-TOF-MS spectra of **21**

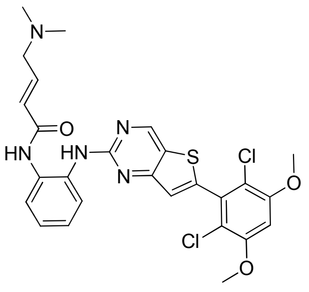

21

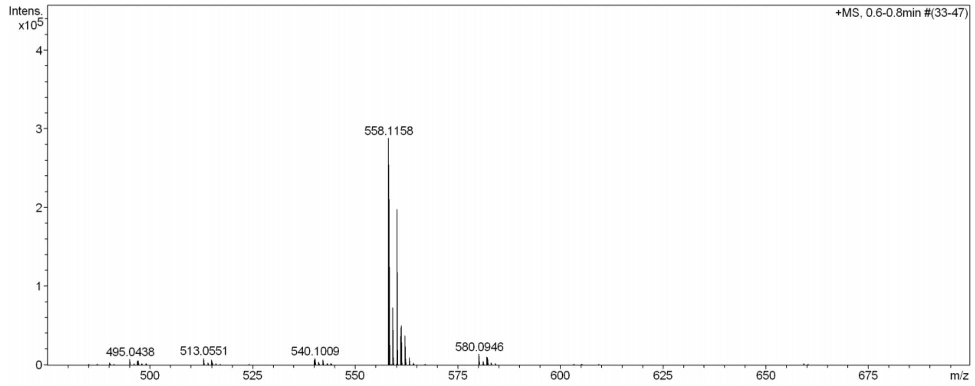

Meas. m/z	#	Formula	m/z	err [ppm]	Mean err [ppm]	rdb	N-Rule	e⁻ Conf	mSigma	Std I	Std Mean m/z	Std I VarNorm	Std m/z Diff	Std Comb Dev
558.1158	1	C 26 H 26 Cl 2 N 5 O 3 S	558.1128	-5.5	-4.3	15.5	ok	even	34.75	0.0392	0.0026	0.0098	0.0015	0.7946

ESI-TOF-MS spectra of **22**

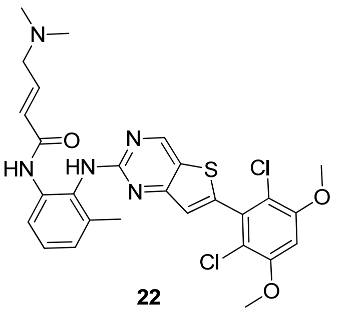

22

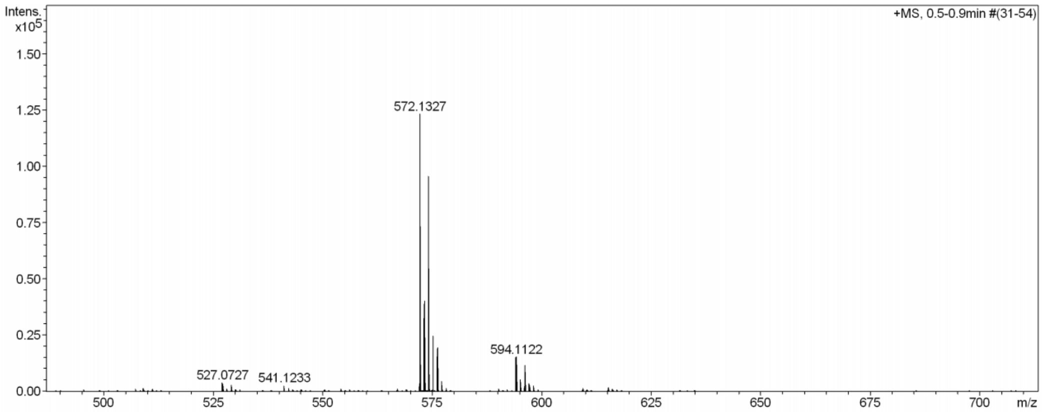

Meas. m/z	#	Formula	m/z	err [ppm]	Mean err [ppm]	rdb	N-Rule	e⁻ Conf	mSigma	Std I	Std Mean m/z	Std I VarNorm	Std m/z Diff	Std Comb Dev
572.1327														
	1	C 27 H 28 Cl 2 N 5 O 3 S	572.1284	-7.4	-7.1	15.5	ok	even	17.86	0.0188	0.0041	0.0054	0.0014	0.7661

ESI-TOF-MS spectra of **23**

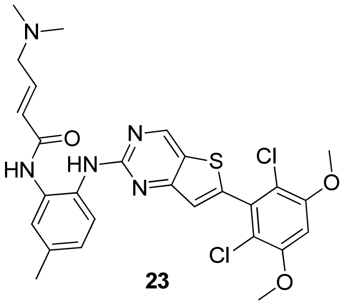

23

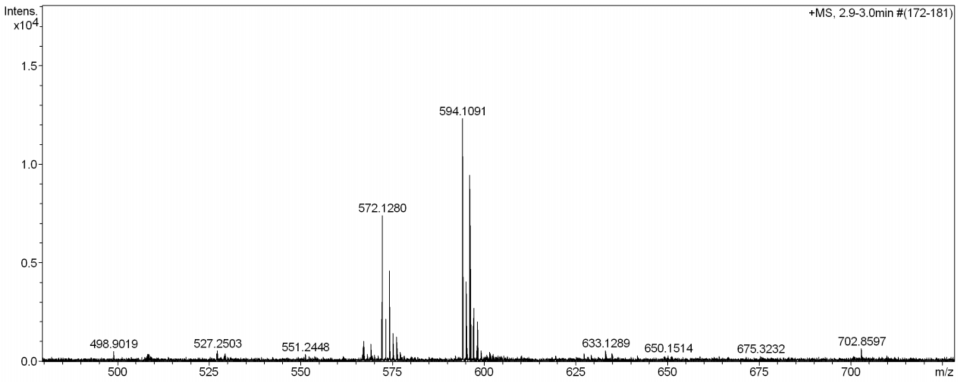

Meas. m/z	#	Formula	m/z	err [ppm]	Mean err [ppm]	rdb	N-Rule	e⁻ Conf	mSigma	Std I	Std Mean m/z	Std I VarNorm	Std m/z Diff	Std Comb Dev
594.1091														

ESI-TOF-MS spectra of **24**

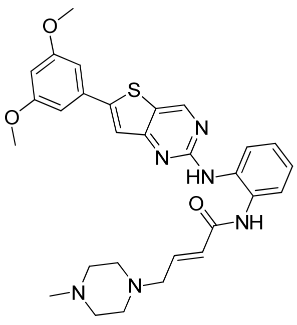

24

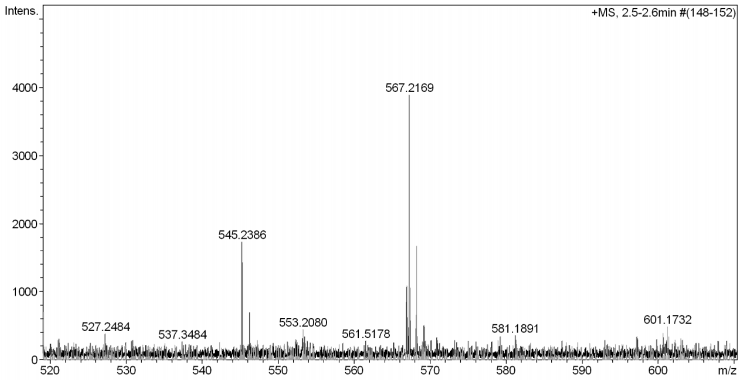

Meas. m/z	#	Formula	m/z	err [ppm]	Mean err [ppm]	rdb	N-Rule	e⁻ Conf	mSigma	Std I	Std Mean m/z	Std I VarNorm	Std m/z Diff	Std Comb Dev
567.2169														
	1	C 29 H 32 N 6 Na O 3 S	567.2149	-3.5	-0.3	16.5	ok	even	44.85	0.0623	0.0030	0.0230	0.0069	0.7284

ESI-TOF-MS spectra of **25**

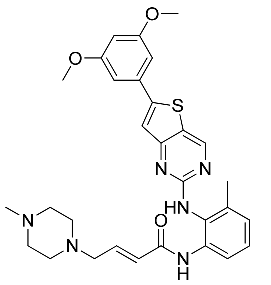

25

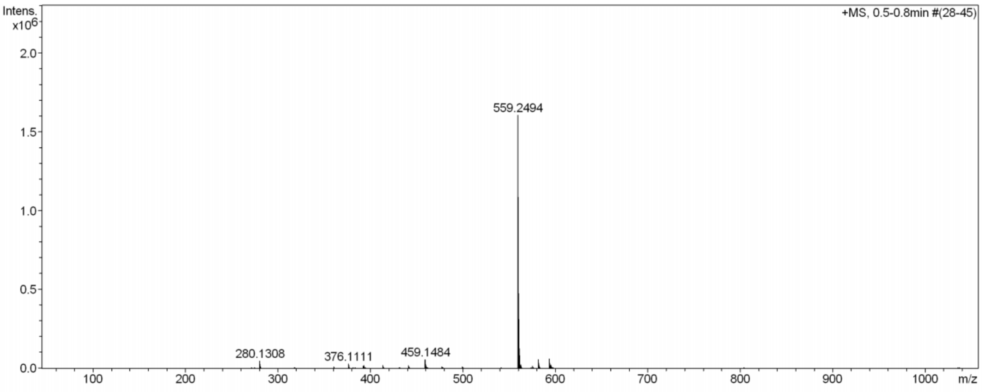

Meas. m/z	#	Formula	m/z	err [ppm]	Mean err [ppm]	rdb	N-Rule	e⁻ Conf	mSigma	Std I	Std Mean m/z	Std I VarNorm	Std m/z Diff	Std Comb Dev
559.2494														
	1	C 30 H 35 N 6 O 3 S	559.2486	-1.5	-1.9	16.5	ok	even	36.76	0.0622	0.0016	0.0210	0.0031	0.8033

ESI-TOF-MS spectra of **26**

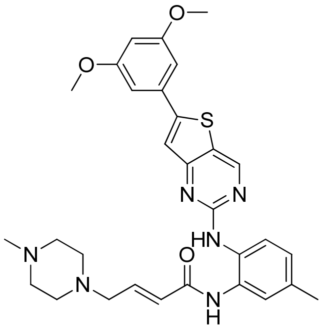

26

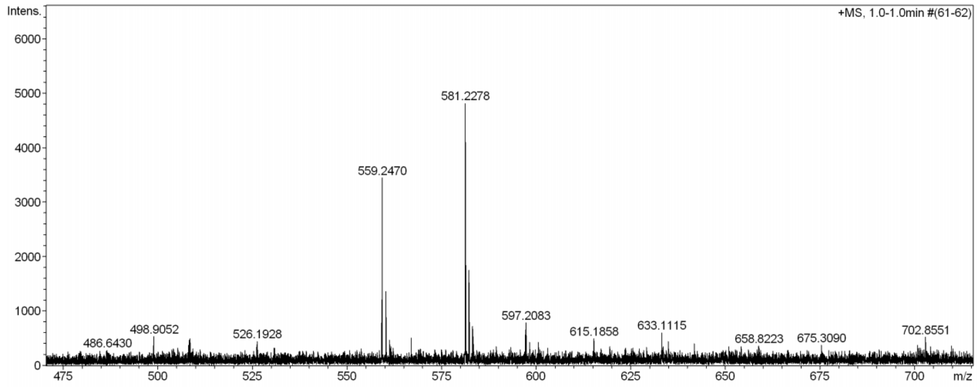

Meas. m/z	#	Formula	m/z	err [ppm]	Mean err [ppm]	rdb	N-Rule	e⁻ Conf	mSigma	Std I	Std Mean m/z	Std I VarNorm	Std m/z Diff	Std Comb Dev
581.2278														
	1	C 30 H 34 N 6 Na O 3 S	581.2305	4.8	3.3	16.5	ok	even	25.28	0.0443	0.0047	0.0207	0.0053	0.7470

ESI-TOF-MS spectra of **27**

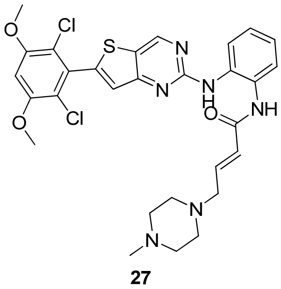

27

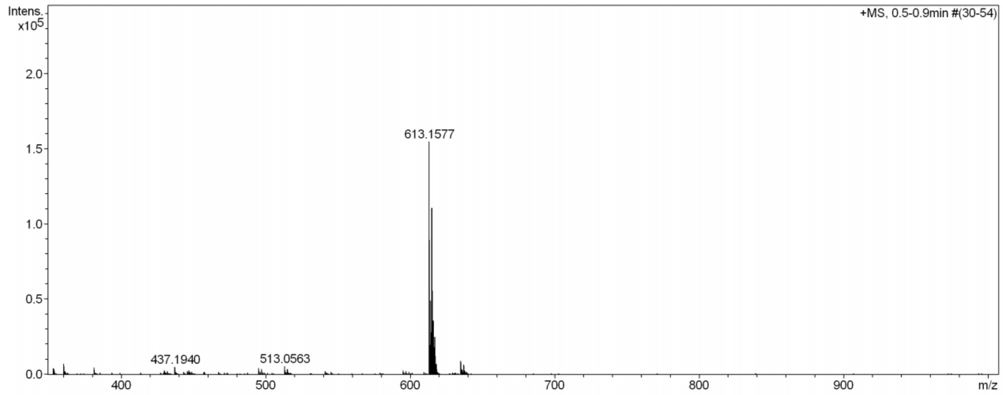

Meas. m/z	#	Formula	m/z	err [ppm]	Mean err [ppm]	rdb	N-Rule	e⁻ Conf	mSigma	Std I	Std Mean m/z	Std I VarNorm	Std m/z Diff	Std Comb Dev
613.1577														
	1	C 29 H 31 Cl 2 N 6 O 3 S	613.1550	-4.5	-4.2	16.5	ok	even	19.45	0.0179	0.0028	0.0044	0.0015	0.6724

ESI-TOF-MS spectra of **28**

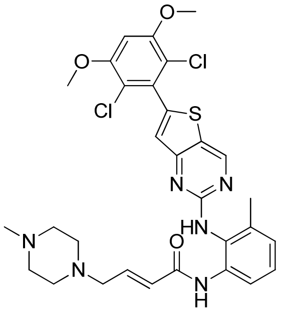

28

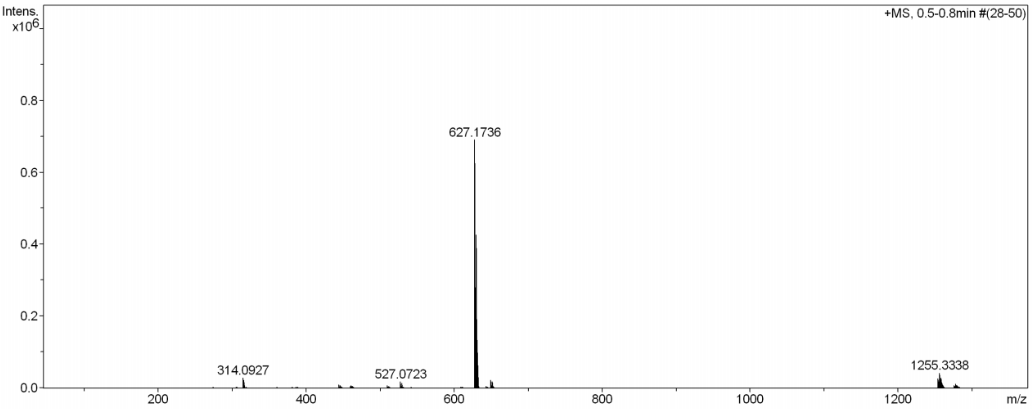

Meas. m/z	#	Formula	m/z	err [ppm]	Mean err [ppm]	rdb	N-Rule	e⁻ Conf	mSigma	Std I	Std Mean m/z	Std I VarNorm	Std m/z Diff	Std Comb Dev
627.1736														
	1	C 30 H 33 Cl 2 N 6 O 3 S	627.1706	-4.8	-4.7	16.5	ok	even	67.09	0.0648	0.0031	0.0156	0.0012	0.8115

ESI-TOF-MS spectra of **29**

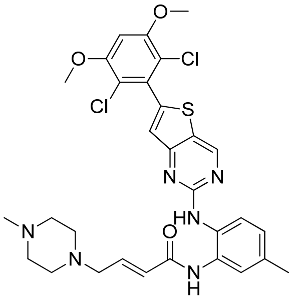

29

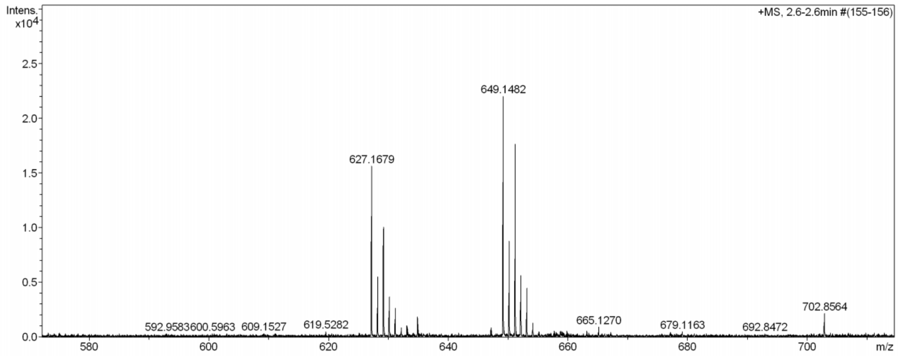

Meas. m/z	#	Formula	m/z	err [ppm]	Mean err [ppm]	rdb	N-Rule	e⁻ Conf	mSigma	Std I	Std Mean m/z	Std I VarNorm	Std m/z Diff	Std Comb Dev
649.1482														

S4. In vitro enzymatic assays of FGFR1 and FGFR4

The effects of compounds on the activities of FGFR kinases were determined using enzyme-linked immunosorbent assay (ELISA) with purified recombinant proteins. Briefly, 20 μg/mL poly (Glu, Tyr)$_{4:1}$ (Sigma, St. Louis, MO, USA) was pre-coated in 96-well plates as a substrate. A 50-μL aliquot of 10 μmol/L ATP solution diluted in kinase reaction buffer (50 mmol/L HEPES [pH 7.4], 50 mmol/L $MgCl_2$, 0.5 mmol/L $MnCl_2$, 0.2 mmol/L Na_3VO_4, and 1 mmol/L DTT) was added to each well; 1 μL of various concentrations of compounds diluted in 1% DMSO (*v*/*v*) (Sigma) were then added to each reaction well. DMSO (1%, *v*/*v*) was used as the negative control. The kinase reaction was initiated by the addition of purified tyrosine kinase proteins diluted in 49 μL of kinase reaction buffer. After incubation for 60 min at 37 °C, the plate was washed three times with phosphate-buffered saline (PBS) containing 0.1% Tween 20 (T-PBS). Anti-phosphotyrosine (PY99) antibody (100 μL; 1:500, diluted in 5 mg/mL BSA T-PBS) was then added. After a 30-min incubation at 37 °C, the plate was washed three times, and 100 μL horseradish peroxidase-conjugated goat anti-mouse IgG (1:2000, diluted in 5 mg/mL BSA T-PBS) was added. The plate was then incubated at 37 °C for 30 min and washed 3 times. A 100-μL aliquot of a solution containing 0.03% H_2O_2 and 2 mg/mL *o*-phenylenediamine in 0.1 mol/L citrate buffer (pH 5.5) was added. The reaction was terminated by the addition of 50 μL of 2 mol/L H_2SO_4 as the color changed, and the plate was analyzed using a multi-well spectrophotometer (SpectraMAX 190, Molecular Devices, Palo Alto, CA, USA) at 490 nm. The inhibition rate (%) was calculated using the following equation: $[1 - (A_{490}/A_{490\ control})] \times 100\%$. The IC_{50} values were calculated from the inhibition curves in two separate experiments.

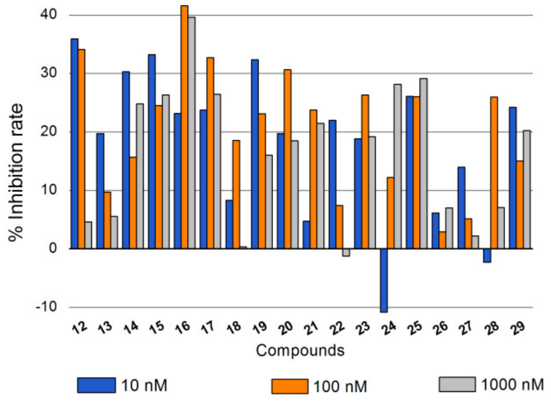

Figure S1. FGFR1 inhibition rate of **12-29** at the concentration of 10 nM, 100 nM and 1000 nM

Table S1. Positive controls in FGFR1 enzymatic assay

Compd.	IC_{50} ± SD（nM）
	FGFR1
**BGJ398**	0.3 ± 0.1

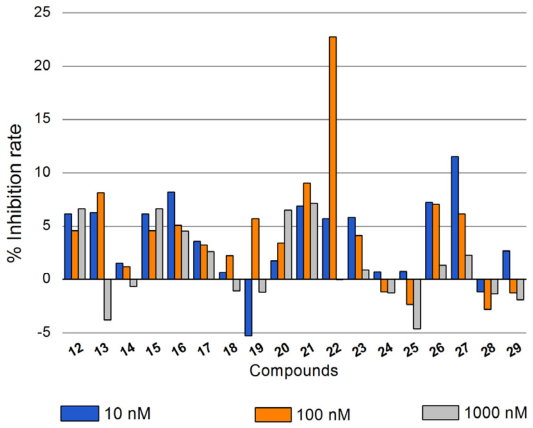

Figure S2. FGFR4 inhibition rate of **12-29** at the concentration of 10 nM, 100 nM and 1000 nM

Table S2. Positive controls in FGFR4 enzymatic assay

Compd.	$IC_{50} \pm SD$（nM）
	FGFR4
LY2874455	3.3±0.1

S5. General procedure for In vitro enzymatic assay of BTK

The HTRF kinEASE TK kit (Cisbio, Codolet, France) was introduced to measure BTK kinase activity and its inhibition by testing compounds at single concentration in duplicate at Medicilon (Shanghai, China). Briefly, compounds at 10 (or 100, 500) nM was incubated with a human recombinant BTK (Merck Millipore, Billerica, MA) and TK substrate-biotin (1 μM) for 10 min at room temperature (RT). ATP (50 μM) was added and then incubated for 50 min at RT. The reaction was stopped by the addition of streptavidin-XL665 (60 nM) and TK antibody-Cryptate (1:100 dilution). After incubation for 60 min at RT, Detection was determined on a PerkinElmer Envision (PerkinElmer Life and Analytical Sciences, Waltham, MA) with 320 nm excitation and emission at 665 and 615 nm. The inhibition rate (%) was analyzed by GraphPad Prism 5.0 (n=2).

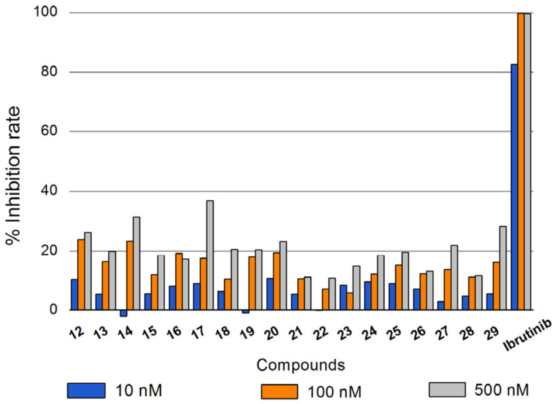

Figure S3. BTK inhibition rate of **12-29** at the concentration of 10 nM, 100 nM and 1000 nM

S6. In vitro enzymatic screen assays

The in vitro enzymatic screen assays of compound **12** were evaluated by ELISA.

Table S3. Inhibition rates of **12** against twenty selected tyrosine kinases

	RET	c-Kit	Flt-3	VEGFR-1	VEGFR-2	VEGFR-3	PDGFR-α	PDGFR-β
**12 (10 μM)**	19.7	0	7.2	0.8	23.1	16.0	0.0	15.5
**12 (1 μM)**	17.8	0	4.2	0.2	12.6	21.5	0.0	10.2
	**EGFR**	**ErbB2**	**ErbB4**	**c-Src**	**ABL**	**EPH-A2**	**IGF1R**	**FAK**
**12 (10 μM)**	0	5.1	12.7	4.3	29.8	5.5	29.5	0.0
**12 (1 μM)**	0	4.4	0	0.0	22.5	2.8	21.2	0.0
	**Fms**	**FGFR2**	**FGFR3**	**EGFR/T790M/L858R**				
**12 (10 μM)**	0	0	0	25.3				
**12 (1 μM)**	0	2.0	0	4.2				

Table S4. Some positive controls in *in vitro* enzymatic screen assays

	**% inhibition rate**		
	**12**	**12**	**Su11248**
	10μM	1μM	1μM
**VEGFR-1**	0.8	0.2	100
**VEGFR-2**	23.1	12.6	98.3
**VEGFR-3**	16	21.5	95.7
**PDGFR-α**	0	0	79.5
**PDGFR-β**	15.5	10.2	95.7
**RET**	19.7	17.8	96.4
**c-Kit**	0	0	88
**Flt-3**	7.2	4.2	91.5
**Fms**	0	0	86.8

Table S5. Some positive controls in *in vitro* enzymatic screen assays

	**% inhibition rate**		
	**12**	**12**	**BIBW2992**
	10μM	1μM	1μM
**EGFR**	0	0	100
**ErbB2**	5.1	4.4	100
**ErbB4**	12.7	0	100
**EGFR/T790M/L858R**	25.3	4.2	99.1

Table S6. Some positive controls in *in vitro* enzymatic screen assays

	**% inhibition rate**			
	**12**	**12**	**Dasatinib**	**PF562271**
	10μM	1μM	1μM	1μM
**c-Src**	4.3	0	95.1	/
**ABL**	29.8	22.5	100	/
**EPH-A2**	5.5	2.8	100	/
**FAK**	0	0	/	93.5

Table S7. Some positive controls in *in vitro* enzymatic screen assays

	**% inhibition rate**			
	**12**	**12**	**AZD4547**	**AEW541**
	10μM	1μM	1μM	1μM
**FGFR2**	0	2	95.4	/
**FGFR3**	0	0	89.4	/
**IGF1R**	29.5	21.2	/	100
